# The impact of COVID-19 on blood donations

**DOI:** 10.1371/journal.pone.0265171

**Published:** 2022-03-24

**Authors:** Besarta Veseli, Sabrina Sandner, Sinika Studte, Michel Clement

**Affiliations:** Institute for Marketing, Hamburg Business School, University of Hamburg, Hamburg, Germany; University of Klagenfurt, AUSTRIA

## Abstract

During a crisis, society calls for individuals to take prosocial actions that promote crisis management. Indeed, individuals show higher willingness to help after a disaster. However, the COVID-19 pandemic presents significant differences as it is an ongoing crisis that affects all individuals and has the potential to pose a direct health threat to anyone. Therefore, we propose that the pandemic may also negatively affect willingness to help, specifically blood donation intentions. It requires a high level of willingness to donate blood beyond the crisis outbreak, as more blood will be needed when postponed surgeries resume. When comparing blood donation intentions from a pre-pandemic study to results from a six-wave (bi-weekly) panel study conducted in Germany during the first pandemic phase (April to June 2020), we find lower medium and long-term blood donation intentions. While active donors show increased awareness of ability and eligibility to donate at the beginning of the pandemic compared to pre-pandemic, they feel significantly less able to donate as the pandemic progresses. Furthermore, inactive donors’ perceived ability to donate significantly decreases in the pandemic phase compared to the pre-pandemic phase. Crucially, both active and inactive donors feel less responsible and less morally obliged to donate, resulting in an overall negative pandemic effect on blood donation intentions. The COVID-19 pandemic compromises blood donations endangering the life-saving blood supply. These alarming results offer evidence-based grounds for practical implications for driving donations in the event of a pandemic.

## Introduction

Societies require prosocial actions by individuals–especially during a crisis. Indeed, individuals are willing to help during disasters, for example, after the 2004 tsunami [1] or September 11, 2001 [2]. Prosocial engagement may even exceed need during disasters in the short term [3], especially as many new donors register [4]. However, a pandemic like COVID-19 is crucially different to other crises. First, contrary to other crises, it has the potential to affect every individual. While in the event of a disaster individuals are likely to feel privileged compared to affected victims, their sense of personal moral obligation to help others might decrease when they themselves are affected by said crisis. Second, COVID-19 is highly transmissible and a threat to one’s own, and others’, health which poses unprecedented challenges to governments, charities, companies, and individuals. Concerning donations, this can especially affect blood donation behavior because of the perceived contagion risk and increasingly challenging conditions (i.e., easing and tightening of restrictions), resulting in decreased perceived ability and eligibility to donate blood. Third, a pandemic is a long-term issue [5, 6], and as such requires continuous willingness to help. This can include monetary support, volunteer assistance, and blood donations. Therefore, in the context of the length of a pandemic, individuals may assess the impact of their donation to be lower than in non-pandemic times. Due to these characteristics of the pandemic, we propose that a pandemic, specifically COVID-19, negatively affects blood donations. We empirically investigate the effect of the COVID-19 pandemic on willingness to donate blood, that is, blood donation intentions. Additionally, we analyze underlying mechanisms relying on pre-pandemic and pandemic data.

In March 2020, crisis management in Germany included measures for the entire population to reduce infections (e.g., social distancing, contact restrictions, and hygiene concepts [4]). These measures were reinforced at the end of April (e.g., wearing face masks in everyday life), and partially relaxed again at the beginning of May 2020 [4, 5]. Blood donation services were faced with the challenge of implementing the imposed hygiene concepts at their donation sites. Consequently, there were fewer blood donation appointments in the first weeks after the measures were set. At the same time, the need for blood initially decreased because surgeries and medical treatments were postponed ensuring hospital capacity for COVID-19-specific treatments [7]. However, in the weeks that followed, blood banks worldwide reported a significant shortage in donations since the pandemic started [8–11]. Crucially, the need for blood is significantly higher in the medium and long term when postponed surgeries are scheduled to take place [12–16]. In fact, approximately 28.4 million planned surgeries (72.3%) worldwide have been postponed due to COVID-19 [17]. This implies a higher demand for blood in the long term. Apart from the existing (in)active donor base, donor recruitment is likely to play a significant role in meeting blood demand. Hence, effective donor management (i.e., retention, reactivation, and recruitment) is crucial, especially because the more time passes since a donation, the less likely a donor is to redonate [18]. Given the longevity of the COVD-19 pandemic, it is imperative to understand its impact: Blood donation intentions might be severely affected even after the pandemic, leading to serious implications for blood banks and society.

Our results show that the COVID-19 pandemic significantly reduces blood donation intentions of both donors and non-donors. Even though blood donation intentions of active blood donors are less affected by COVID-19, the pandemic has an overall negative effect on donation intentions, especially medium and long-term donation intentions decrease. In fact, compared to pre-pandemic, both active and inactive donors feel less responsible and less morally obliged to donate blood. Additionally, our results show that inactive donors perceive their donation to have less impact, compared to pre-pandemic. Moreover, inactive donors feel less able to donate blood. More importantly, active donors’ perceived ability to donate increases at the beginning of the pandemic compared to pre-pandemic but significantly decreases as the pandemic progresses (i.e., end of the first pandemic phase in Germany). We can further show the effect of changes in the underlying mechanisms on donation intentions of active and inactive donors during the pandemic.

We contribute to donation literature and previous research on prosocial behavior during crises by measuring the effect of COVID-19 on short-term, medium-term, and long-term blood donation intentions, and by using pre-pandemic (baseline) as well as pandemic data. Relying on both pre-pandemic and pandemic data, we are able to analyze the effects of an external shock (i.e., the pandemic) compared to a baseline of donation intentions and underlying mechanisms. While previous research focuses on short-term donation behavior in the event of a disaster, little is known on how long-term donation behavior is affected by a pandemic like COVID-19 [19], and specifically, how blood donations are affected. Donating blood is more personal as blood donors give from their own body, and thus, different to other donation types (i.e., donating money or volunteering) [20, 21]. Moreover, contrary to prior literature, we analyze the effect of the COVID-19 pandemic for both donors and non-donors, and distinguish between active donors (i.e., donors who have donated blood at least once in the last 24 months) and inactive donors (i.e., donors who have not donated blood in the last 24 months). Hence, we derive differentiated implications for blood banks.

### Hypotheses development

Given the unprecedented case of a pandemic like COVID-19, there is little knowledge on its effects on blood donations. A pandemic, however, significantly differs from other crises investigated in the literature. First, every individual is affected by the crisis, specifically, by the extent of the crisis and the measures imposed by governments (e.g., restrictions on public and private life). Second, in general, there is a potential direct threat to one’s own, and others’, health. Third, a pandemic differs regarding the time length of the crisis. While disasters are punctual events that are usually both confined to specific times and spaces, a pandemic is an ongoing large-scale crisis. Due to these pandemic characteristics contrasting other crises, we propose that a pandemic can negatively affect blood donations and formally hypothesize the following:

*H1*: *The COVID-19 pandemic decreases blood donation intentions*.

In the event of a disaster, individuals are faced with their own mortality, resulting in increased prosocial acts [22]. This has specifically shown to be true for causes that promote crisis management [22], and for socially conscious consumer behavior [23] in which individuals can suppress thoughts of their own mortality by engaging in prosocial activities [24]. Moreover, individuals are motivated to donate based on the personal feeling that they are morally obliged to do so; thus, an individual’s personal moral norms are significant drivers of their donation intention [25–27]. Recent research has shown that individuals’ personal moral norms have dropped during the current pandemic compared to pre-pandemic [5]. Specifically, individuals feel less responsible and less morally obliged to engage in prosocial activities. As personal moral norms are important predictors of moral behavior [6, 25, 26], a decrease in this could lead to less prosocial engagement. While Veseli et al. [5] analyze how personal moral norms regarding prosocial activities are changing, this work focuses on how blood donation intentions are affected by a pandemic (i.e., COVID-19). It is important to note that donating blood is also different to other donations (i.e., donating money, volunteering) in the sense that blood donors give from their own body, which makes it more personal [20, 21]. Contrary to other crises, a pandemic affects every individual, consequently, every potential blood donor. Thus, while in the case of a disaster individuals might feel more privileged compared to those affected, resulting in higher willingness to donate; we propose that they behave differently in the case of a pandemic. Individuals are required to follow restrictions on their public and private lives (e.g., social distancing, contact restrictions, etc.), and also face the threat of being infected as well as infect others. Therefore, responsibility to act prosocially in one way can attenuate perceived responsibility to act prosocially in other ways. We specifically argue that individuals are likely to feel less personally responsible and morally obliged to help others when they are affected by a crisis themselves, resulting in lower willingness to donate. Thus, we formally hypothesize:

*H2*: *The COVID-19 pandemic decreases blood donation intentions, as the individual’s sense of moral obligation to donate blood, measured by personal moral norms, drops*.

Blood donor motivation literature has demonstrated the importance of self-efficacy in predicting donation intention [e.g., 26, 28, 29]. Self-efficacy is defined as the person’s perceived ability or capability to perform a certain behavior [27, 30]. To be eligible to donate blood requires good health. The self-efficacy construct, therefore, particularly measures an individual’s perceived ability and capability focusing on the factor of health [27]. As remaining healthy is a prerequisite for donating blood, research has shown that donors are motivated to donate blood because they can demonstrate their good health (i.e., healthy donor effect, [31]). However, the COVID-19 pandemic, in contrast to other crises, poses a direct contagion risk to all individuals, potentially threatening one’s own, and others’, health. Therefore, we argue that in the case of a pandemic individuals may feel less able to donate and find it more difficult to do so despite being healthy, that is, self-efficacy can decrease, resulting in lower willingness to donate. We formally hypothesize:

*H3*: *The COVID-19 pandemic decreases blood donation intentions as the individual’s perceived ability to donate blood, measured by self-efficacy, drops*.

Additionally, the impact that donors expect from their contribution is an important driver of charitable behavior [32]. Research has found that the probability to donate decreases when potential donors perceive their donation does not make a difference [32–34]. Theorization on effective altruism [35] suggests that individuals choose to donate based on welfare maximization (i.e., donate to charities with the highest impact). Concerning the concept of effective altruism, concepts of impact philanthropy [32] and perceived impact [36, 37] concern a donor’s personal expectation and assessment of how impactful their contribution will be. A pandemic is a long-term crisis and while donating during a crisis is likely to be effective and increase welfare, we argue that individuals are likely to expect their contribution to help less in the context of the crisis’ length. During a pandemic, individuals are likely to assess the impact of their donation to be less compared to the perceived impact of their donation during non-pandemic times. We propose that a long-term, worldwide crisis, like the current pandemic, can decrease an individual’s perceived impact of their donation, leading to a decrease in willingness to donate. We formally hypothesize:

*H4*: *The COVID-19 pandemic decreases blood donation intentions as the individual’s perceived impact of their donation drops*.

## Materials and methods

The Dean of Research of the Business School of University of Hamburg reviewed and approved this research proposal twofold with respect to ethics–before the data was collected in April 2019 and in April 2020.

Participants of both surveys were invited by respondi AG–a professional market research company, which provides an online access panel. Respondents need to agree to participate in surveys with respondi (i.e., participant consent) and each survey is reviewed by the company before the survey is sent to participants. Thus, the market research company manages participant consent.

We conducted two studies–an online study in April 2019 (n = 2,449) and a panel study consisting of six waves (t_1_ to t_6_) carried out biweekly from April to June 2020 (sample size ranges from n_1_ = 1,499 to n_6_ = 818, S1 Table in S1 File). Both studies are based on a demographically representative sample of the German population. We define data from April 2019 as the pre-pandemic phase–our baseline, and data from April to June 2020 as the pandemic phase–capturing the effect of COVID-19. In both studies, we asked participants to provide demographics and information on donation history (i.e., donor or non-donor) as well as donor status (i.e., active or inactive). Applying Red Cross classification, we define active donors as donors who have donated blood at least once in the last 24 months, while inactive donors are defined as blood donors who have not donated in the last 24 months [38]. Non-donors are defined as individuals who have never donated blood in their lives. We account for donation history and donor status as prior research has shown that there are systematic differences between blood donors and other individuals (e.g., blood donors are more willing to engage in various types of prosocial behavior [39]). Furthermore, the time period since the last donation is likely to affect donation intentions, that is, inactive donors are likely to behave differently in times of crises compared to active donors. Indeed, research has shown that the more time passes since a donation, the less likely a donor is to redonate [18], and active donors who are more familiar with a cause may show increased motivation to help [40].

We measured blood donation intentions (“I intend to donate blood …,” 1 = strongly disagree to 7 = strongly agree) in the short term (“…on the next possible date”), medium term (“…over the next six months”), and long term (“It is likely that I will donate blood in the future”) as our dependent variables [26]. Measuring donation intentions allows us to derive predictions and implications for blood donation behavior aside from short-term blood donation behavior. Due to the systematic cap in the first pandemic phase (i.e., limited blood collection events and imposed mobility restrictions), initial (i.e., short-term) donation behavior does not reflect what is needed in the medium and long term when postponed surgeries take place. In addition to blood donation intentions, we measured personal moral norms regarding blood donation using four items: (1) “I feel a personal responsibility to give blood,” (2) “I feel a moral obligation to give blood,” (3) “I feel a social obligation to give blood,” (4) “Sometimes I feel guilty that I do not donate blood” [26], self-efficacy using three items: (1) “If I wanted to, I would be able to give blood as long as my health allows it,” (2) “I think myself capable of continuing to give blood as long as my health allows it,” (3) “I find it hard to give blood time after time” [27], as well as perceived impact using three items: (1) “My blood donation is needed,” (2) “My blood donation makes a difference,” (3) “My blood donation has an impact” [37]. All three constructs are measured on a 7-point-scale (1 = strongly disagree to 7 = strongly agree; mean values and standard deviations provided in S3-S5 Tables in S1 File).

Since we rely on two independent studies for our analysis (i.e., April 2019 online study and April-June 2020 panel study), we checked for systematic differences. As respondents in the two studies differ in terms of gender, donation history, and donor status (S1 Table in S1 File), we account for these systematic differences in our samples by weighting the data in the pandemic phase by gender, donation history, and donor status with 2019 as the baseline distribution (S1 Table in S1 File). We use the weighted data for pandemic to pre-pandemic mean value comparisons.

In our panel study analysis, we account for panel mortality by relying on balanced data in our panel model, that is, only including respondents who completed all six waves of the study in the analysis (i.e., 593 respondents). We further control for age and gender in our model since these respondents differ from those who did not participate in all waves (S2 Table in S1 File). To measure the effect of changes in the underlying mechanisms on donation intention over time, we used a fixed effects panel estimator with a first-difference approach to eliminate individual effects. This approach controls for unobservable fixed effects as well as for unobservable (time-invariant) differences between individuals [41]. In addition to the underlying mechanisms, we include the following variables in the panel model to control for pandemic-specific influences: (1) concern (i.e., “I am mostly concerned.” 7-point-scale, 1 = strongly disagree to 7 = strongly agree), (2) expected return to everyday life (i.e., “When do you think you can return to your everyday life?” 1 = “in the next two weeks” to 7 = “in more than a year”), (3) informed (i.e., “I feel sufficiently informed about the issue of blood donations during the Corona crisis.” 7-point-scale, 1 = strongly disagree to 7 = strongly agree), (4) trust in COVID-19 measures at blood collections (i.e., “To what extent do you trust the collection center with regard to (a) the hygiene precautions and (b) the distance regulation?” 7-point-scale, 1 = strongly disagree to 7 = strongly agree), (5) SARS-CoV-2 infection (i.e., “Have you been tested positive for SARS-CoV-2?” 0 = no, 1 = yes).

## Results

### Donation intentions

**Fig 1:**
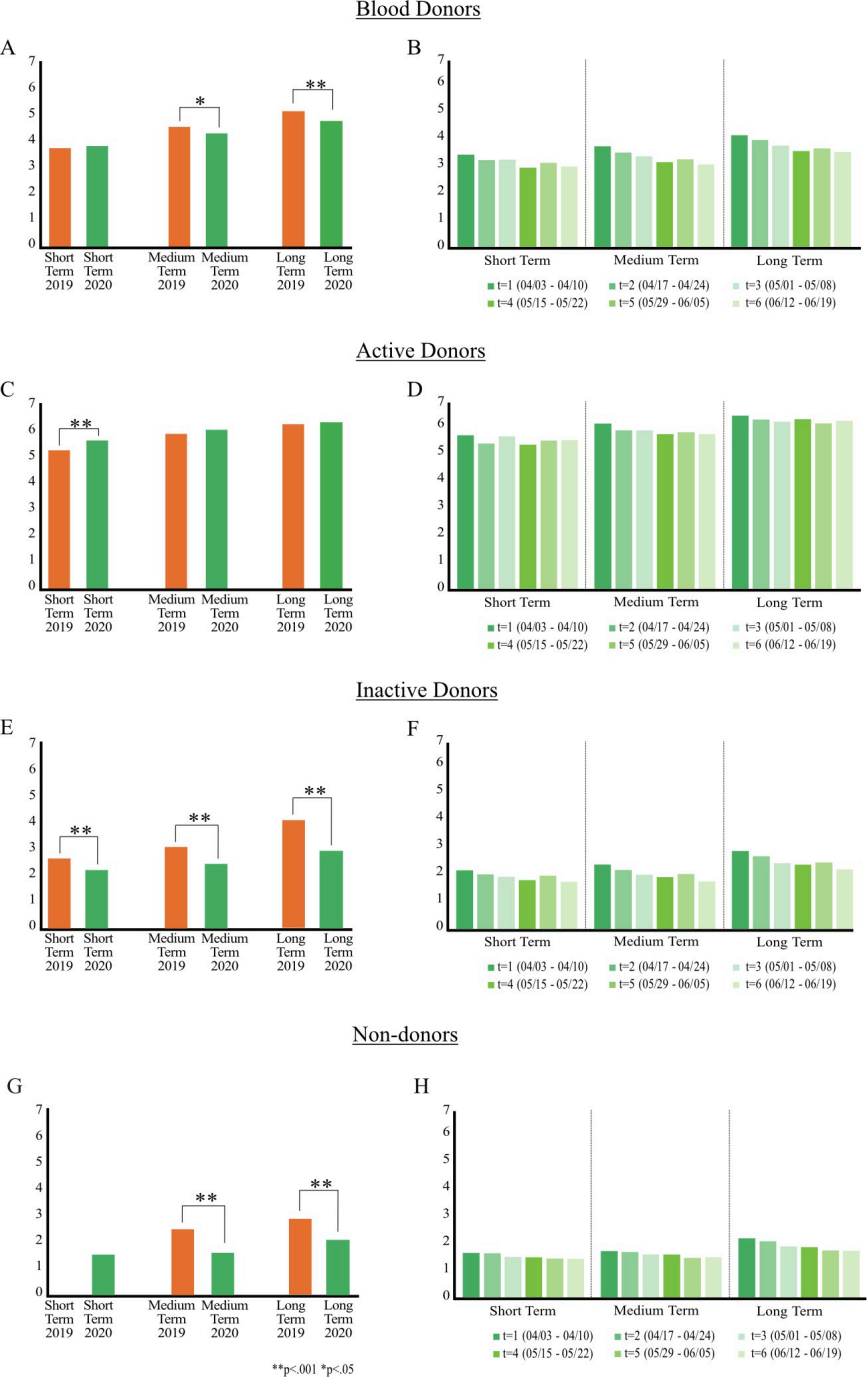
Effect of COVID-19 on blood donation intentions. (A) Comparison of reported mean values by blood donors (overall) between pandemic phase (t_1_, 2020/04) and pre-pandemic (2019/04). Significant changes are marked. (B) Reported mean values by blood donors (overall) within the pandemic phase (t_1_ to t_6_). (C) Same as (A) only for active donors. (D) Same as (B) only for active donors. (E) Same as (A) only for inactive donors. (F) Same as (B) only for inactive donors. (G) Same as (A) only for non-donors. (H) Same as (B) only for non-donors.

We compare donation intentions to investigate whether the pandemic affects blood donations. We conduct our analysis in two steps. First, we provide model-free evidence based on t-tests by comparing the pre-pandemic and the beginning of the pandemic phase (t_1_), relying on the weighted data as described before. Overall, donation intentions of blood donors (i.e., active and inactive) are significantly lower in t_1_ compared to pre-pandemic (Fig 1A, S8 Table in S1 File). In the short term, donation intentions of active donors increase in t_1_ (M = 5.72, SD = 1.76) compared to pre-pandemic (M = 5.36, SD = 1.71, t(1029) = 3.295, p = .001, Fig 1C). By contrast, donation intentions of inactive donors are significantly lower in t_1_ compared to pre-pandemic in the short term (M = 2.00, SD = 1.64 vs. M = 2.38, SD = 1.71, t(1029) = -3.509, p < .001, Fig 1E), medium term (M = 2.20, SD = 1.85 vs. M = 2.78, SD = 1.96, t(1029) = -4.722, p < .001), and long term (M = 2.65, SD = 2.14 vs. M = 3.67, SD = 2.29, t(1029) = 7.089, p < .001). Likewise, donation intentions of non-donors are significantly lower at the beginning of the pandemic (t_1_) compared to pre-pandemic (Fig 1G).

To measure the effect of the pandemic for active and inactive donors, we conduct regression analyses controlling for gender and donor status (Table 1, Model 1–3). Consistent with our hypothesis H1, results confirm that the pandemic has a significant negative main effect on blood donation intentions in the short term (Table 1, Model 1), medium term (Table 1, Model 2), and long term (Table 1, Model 3). However, we find a positive interaction effect between the pandemic effect and donor status (Table 1, Model 1–3). Thus, the negative pandemic effect is smaller for active donors. Short-term and medium-term donation intentions of active donors compared to inactive donors increase (Table 1, Model 1–2), while long-term donation intentions decrease (Table 1, Model 3). Moreover, results show that active donors report significantly higher donation intentions compared to inactive donors (Table 1, Model 1–3).

**Table 1:** Results of regression analysis measuring the effect of the pandemic.

	Active and Inactive Donors									Donors and Non-donors					
	Model 1			Model 2			Model 3			Model 4			Model 5		
	Short-term donation intentions			Medium-term donation intentions			Long-term donation intentions			Medium-term donation intentions			Long-term donation intentions		
Predictor	β	CI	*p*	β	CI	*p*	β	CI	*p*	β	CI	*p*	β	CI	*p*
Pandemic effect	**-.38**	**-.58-(-.17)**	***<* .*001***	**-.57**	**-.77-(-.36)**	***<* .*001***	**-.99**	**-1.2-(-.77)**	***<* .*001***	**-.76**	**-.93-(-.59)**	***<* .*001***	**-.94**	**-1.12-(-.76)**	***<* .*001***
*Interaction 1*															
Pandemic effect x donor status	**.67**	**.34-.99**	***<* .*001***	**.58**	**.25-.91**	**.*001***	**.86**	**.52–1.21**	***<* .*001***						
*Interaction 2*															
Pandemic effect x donation history										-.08	-.33-.17	.*516*	-.13	-.39-.13	.*317*
Donor status^a^	**2.93**	**2.74–3.13**	***<* .*001***	**3.06**	**2.87–3.25**	***<* .*001***	**2.44**	**2.24–2.64**	***<* .*001***						
Donation history^b^										**2.05**	**1.90–2.21**	***<* .*001***	**2.09**	**1.93–2.25**	***<* .*001***
Age	-**.01**	**-.01-(-.00)**	**.*027***	**-.02**	**-.03-(-.02)**	***<* .*001***	**-.04**	**-.04-(-.03)**	***<* .*001***	**-.04**	**-.04-(-.04)**	***<* .*001***	**-.06**	**-.06-(-.05)**	***<* .*001***
Gender^c^	-.02	-.17-.13	.*783*	-.06	-.21-.09	.*446*	.01	-.15-.17	.*889*	.06	-.06-.18	.*313*	.08	-.04-.20	.*207*
Intercept	2.71	2.39–3.04	*<* .*001*	3.94	3.62–4.27	*<* .*001*	5.57	5.23–5.91	*<* .*001*	4.19	3.95–4.43	*<* .*001*	5.54	5.29–5.79	*<* .*001*
N	1,964			1,964			1,964			3,948			3,948		
R^2^	.474			.523			.473			.288			.323		

Significant results are marked in bold.

^a^(1: active donor / 0: inactive donors)

^b^(0: non-donor / 1: donor)

^c^(0: male / 1: female / 2: others)

We also examine the pandemic effect for donors and non-donors using regression analyses (Table 1, Model 4–5). Results confirm hypothesis H1: The pandemic has a significant negative effect on blood donation intentions in the medium term (Table 1, Model 4) and long term (Table 1, Model 5; Short-term donation intentions of non-donors were not reported in the pre-pandemic phase). The interaction effect between the pandemic effect and donation history (i.e., donor or non-donor) is not significant (Table 1, Model 4–5). Therefore, not differentiating between active and inactive donors, the pandemic effect is not moderated by donation history. However, donors report significantly higher donation intentions compared to non-donors (Table 1, Model 4–5).

Second, we analyze the development of donation intentions within the pandemic (t_1_ to t_6_) capturing a time frame of 12 weeks. As we rely on repeated data measures within the pandemic, we use within-subjects ANOVAs and adjust the p-values using the Bonferroni multiple testing correction method. Not distinguishing between donor status, the pandemic (t_1_ to t_6_) does significantly influence long-term donation intentions of blood donors over time (*p*_*adj*_ *<* .*001*, S10 Table in S1 File), that is, long-term donation intentions decrease as the pandemic progresses (Fig 1B). Reported short-term, medium-term, and long-term donation intentions of active donors do not significantly change as the pandemic progresses (Fig 1D, S10 Table in S1 File). However, the pandemic does significantly affect long-term donation intentions of inactive donors over time (*p*_*adj*_ *=* .*016*, S10 Table in S1 File), which decrease during the pandemic (Fig 1F). More importantly, donation intentions of active compared to inactive donors significantly differ in the short term (e.g., M_t1_ = 5.67, SD_t1_ = 1.78 vs. M_t1_ = 2.00, SD_t1_ = 1.63, diff = 3.678[3.247–4.110], p < .001), medium term (e.g., M_t1_ = 6.11, SD_t1_ = 1.49 vs. M_t1_ = 2.19, SD _t1_ = 1.85, diff = 3.914[3.475–4.353], p < .001), and long term (e.g., M_t1_ = 6.40, SD_t1_ = 1.28 vs. M_t1_ = 2.65, SD_t1_ = 2.13, diff = 3.754[3.276–4.232], p < .001; S11 Table in S1 File). We also observe that the pandemic significantly influences donation intentions of non-donors in the short term, medium term, and long term over time, that is, donation intention drop further during the pandemic (Fig 1H, S10 Table in S1 File). These results provide additional support in favor of H1.

Overall, our results confirm hypothesis H1. However, the regression results highlight that the size of the pandemic effect on donation intentions is smaller for active donors compared to inactive donors. Short-term and medium-term donation intentions of active donors increase compared to pre-pandemic, contrary to H1. However, long-term donation intentions of active donors significantly decrease, supporting H1. In line with H1, donation intentions of inactive donors and non-donors further decrease during the pandemic.

### Underlying mechanisms

We first analyze whether the underlying mechanisms personal moral norms (PMN), self-efficacy (SE), and perceived impact (PI) of donors have changed compared to pre-pandemic using t-tests. (We did not measure underlying mechanisms for non-donors.) In addition, we use within-subjects ANOVAs and Bonferroni’s multiple testing correction method for adjusting the p-values to reveal the development of the underlying mechanisms during the pandemic. Second, to test our hypotheses H2 to H4, we use mediation analysis to investigate the direct effect of the pandemic and the indirect effects through personal moral norms, self-efficacy, and perceived impact. Third, we conduct a panel regression analysis to further estimate the effects of changes in our independent variables on donation intentions over time.

**Fig 2:**
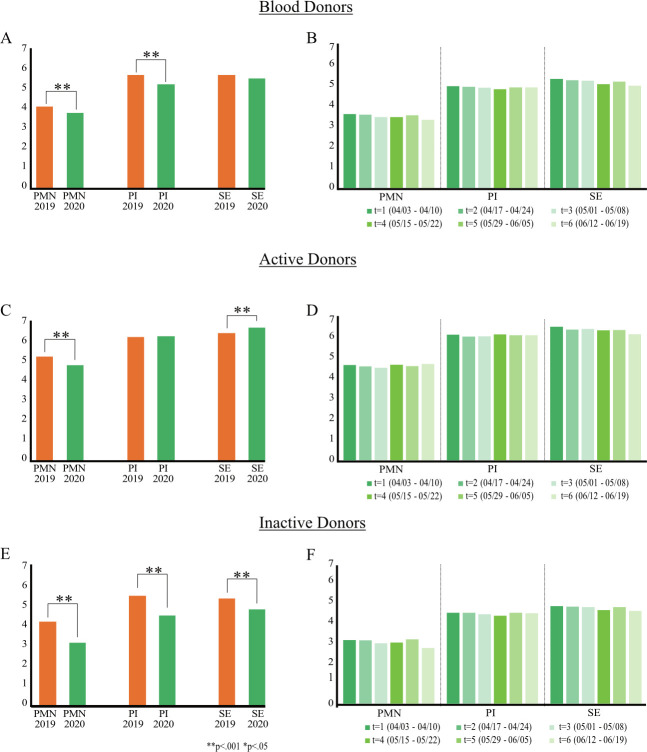
Effect of COVID-19 on blood donor motivations. (A) Comparison of reported mean values of personal moral norms (PMN), perceived impact (PI), and self-efficacy (SE) by blood donors (overall) between pandemic phase (t_1_, 2020/04) and pre-pandemic phase (2019/04). Significant changes between waves are marked. (B) Reported mean values by blood donors within the pandemic phase (t_1_ to t_6_). (C) Same as (A) only for active donors. (D) Same as (B) only for active donors. (E) Same as (A) only for inactive donors. (F) Same as (B) only for inactive donors.

Overall, blood donors report significantly lower levels of personal moral norms and perceived impact at the beginning of the pandemic (t_1_) compared to pre-pandemic (Fig 2A, S9 Table in S1 File). In contrast, reported values of self-efficacy do not significantly change (Fig 2A). However, results differ between active and inactive donors. While self-efficacy of active donors has increased (M = 6.45, SD = .79 vs. M = 6.21, SD = .93, t(1029) = 4.303, p < .001, Fig 2C), inactive donors report lower values in t_1_ compared to pre-pandemic (M = 4.66, SD = 1.82 vs. M = 5.17, SD = 1.51, t(1029) = -4.804, p<0.001, Fig 2E). Personal moral norms of active donors (M = 4.65, SD = 1.58 vs. M = 5.08, SD = 1.31, t(1029) = -4.687, p < .001) and inactive donors (M = 3.04, SD = 1.73 vs. M = 4.05, SD = 1.65, t(1029) = -9.337, p<0.001) are lower at the beginning of the pandemic (t_1_) compared to pre-pandemic. Regarding perceived impact, the reported values of active donors do not significantly change, whereas inactive donors report lower values in t_1_ compared to pre-pandemic (M = 4.35, SD = 2.05 vs. M = 5.31, SD = 1.59, t(1029) = -8.357, p<0.001, Fig 2E).

Analyzing the development of the underlying mechanisms within the pandemic (t_1_ to t_6_) using within-subjects ANOVAs, the pandemic (t_1_ to t_6_) does significantly influence self-efficacy of active donors over time (*p*_*adj*_ *=* .*036*, S12 Table in S1 File), that is, self-efficacy decreases as the pandemic progresses (Fig 2D). However, personal moral norms and perceived impact of active donors do not change as the pandemic progresses (t_1_ to t_6_). The same applies for inactive donors; personal moral norms, perceived impact, and self-efficacy do not significantly change as the pandemic progresses (Fig 2F, S12 Table in S1 File). We do observe significant differences between active and inactive donors. Active donors report significantly higher mean values of personal moral norms (e.g., M_t1_ = 4.60, SD_t1_ = 1.60 vs. M_t1_ = 3.04, SD_t1_ = 1.72, diff = 1.566 [1.102–2.029], p < .001), perceived impact (e.g., M_t1_ = 6.06, SD_t1_ = 1.00 vs. M_t1_ = 4.35, SD_t1_ = 4.34, diff = 1.713 [1.233–2.194], p < .001), and self-efficacy (e.g., M_t1_ = 6.44, SD_t1_ = .81 vs. M_t1_ = 4.66, SD_t1_ = 1.82, diff = 1.779 [1.367–2.191], p < .001) within the pandemic compared to inactive donors (S13 Table in S1 File).

Given the significant decrease in blood donation intentions in the medium and long term for blood donors, we analyze the underlying mechanisms conditional on donor status using mediation analysis [42]. We compare t_1_ of pandemic to pre-pandemic and focus on donation intentions for the medium term (i.e., next six months) as willingness to donate blood is crucial in resuming the postponed surgeries in the months after the crisis outbreak (Table 2). Results confirm that donor status positively moderates the negative pandemic effect on personal moral norms, perceived impact, and self-efficacy. The pandemic effect on reported donor motivations is therefore less negative for active donors. The interaction effect between the pandemic phase in t_2_ to t_6_ and donor status is also positive and significant (S17–S31 Tables in S1 File). More importantly, results show that the negative pandemic effect for active donors is mediated by a drop in personal moral norms, despite a positive mediated effect through self-efficacy. Self-efficacy, however, is only a significant mediator in t_1_. Although the effect size of the mediation through personal moral norms on blood donation intentions for active donors decreases during the pandemic (b = -.18 in t_1_ to b = -.03 in t_6_, S14-S31 Tables in S1 File), it remains significant and supports H2. However, contrary to H3, we find no significant mediated effect through perceived impact on donation intentions of active donors. By contrast, the decrease in donation intentions of inactive donors is mediated by a drop in all three donor motivations throughout all six waves, confirming H2, H3, and H4. Detailed statistical results are provided in the (S14-S31 Tables and S2 Fig in S1 File). Overall, these results confirm H2 for both active and inactive donors, and H3 and H4 for inactive donors.

**Table 2:** Results of the moderated mediation analysis on donation intentions in the medium term regarding pre-pandemic and pandemic (t = 1).

	Personal Moral Norms				Perceived Impact				Self-Efficacy				Donation Intention^2^			
	b (se)	*p*	2.5%	97.5%	b (se)	*p*	2.5%	97.5%	b (se)	*p*	2.5%	97.5%	b (se)	*p*	2.5%	97.5%
Pandemic Effect	**-1.013 (.096)**	**< .001**	**-1.201**	**-.824**	**-.960 (.092)**	**< .001**	**-1.141**	**-.780**	**-.505 (.084)**	**< .001**	**-.670**	**-.341**	-.077 (.093)	.409	-.260	.106
Donor Status (DS)	**1.029 (.087)**	**< .001**	**.858**	**1.200**	**.694 (.084)**	**< .001**	**.530**	**.859**	**1.046 (.076)**	**< .001**	**.896**	**1.195**				
Pandemic Effect × DS	**.537 (.153)**	**.001**	**.236**	**.837**	**1.019 (.147)**	**< .001**	**.731**	**1.308**	**.733 (.134)**	**< .001**	**.471**	**.996**				
Personal Moral Norms (PMN)													**.381 (.030)**	**< .001**	**.322**	**.440**
Perceived Impact (PI)													**.210 (.032)**	**< .001**	**.147**	**.272**
Self-Efficacy (SE)													**.601 (.032)**	**< .001**	**.538**	**.664**
Constant	**4.048 (.062)**	**< .001**	**3.927**	**4.169**	**5.305 (.059)**	**< .001**	**5.189**	**5.421**	**5.165 (.054)**	**< .001**	**5.059**	**5.271**	**-1.952 (.193)**	**< .001**	**-2.33**	**-1.574**
Conditional Indirect Effect via PMN																
DS^1^ (0)													**-.386 (.052)**		**-.492**	**-.285**
DS (1)													**-.181 (.045)**		**-.273**	**-.094**
Conditional Indirect Effect via PI																
DS (0)													**-.201 (.039)**		**-.280**	**-.129**
DS (1)													.012 (.017)		-.020	.045
Conditional Indirect Effect via SE																
DS (0)													**-.303 (.064)**		**-.430**	**-.182**
DS (1)													**.137 (.040)**	** **	**.059**	**.215**
N	1,964				1,964				1,964				1,964			
R^2^	.195				.160				.201				.408			
df	3.000				3.000				3.000				4.000			
F (*p*)	158.059	< .001			124.629	< .001			163.933	< .001			337.633	< .001		

Significant results are marked in bold.

^1^0 = Inactive donors 1 = active donors

^2^ “I intend to donate blood over the next six months”.

Drawing on the panel data structure, we further analyze donation intentions and underlying mechanisms within the pandemic over time. To specifically measure how changes in the underlying mechanisms can affect donation intentions during the pandemic (t_1_ to t_6_), we estimate a panel regression using a first-difference approach, thereby the estimator is specified after first-differencing the equation and is based on the within-transformation. Using this approach, we can control for both unobservable fixed effects and unobservable differences between individuals [41]. Although we control for age and gender, the respective effects are not displayed as these are time-fixed effects. Detailed statistical results as well as the correlation matrices are provided in the (S4-S7 Tables in S1 File). Table 3 shows the results for active donors (Model 1–3) and inactive donors (Model 4–6).

**Table 3:** Panel regression analysis for active and inactive donors.

	Active Donors									Inactive Donors								
	Model 1			Model 2			Model 3			Model 4			Model 5			Model 6		
	Short-term donation intentions			Medium-term donation intentions			Long-term donation intentions			Short-term donation intentions			Medium-term donation intentions			Long-term donation intentions		
Predictor	β	CI	*p*	β	CI	*p*	β	CI	*p*	β	CI	*p*	β	CI	*p*	β	CI	*p*
Self-efficacy	**.24**	**.06-.43**	**.*011***	**.37**	**.19-.55**	***<* .*001***	**.36**	**.23-.50**	***<* .*001***	**.06**	**.00-.11**	**.*040***	**.09**	**.03-.15**	**.*005***	**.07**	**.00-.13**	**.*042***
Personal moral norms	**.19**	**.02-.36**	**.*032***	**.31**	**.15-.48**	***<* .*001***	**.14**	**.02-.26**	**.*026***	**.07**	**.01-.13**	**.*034***	**.10**	**.03-.16**	**.*007***	**.10**	**.02-.17**	**.*011***
Perceived impact	**.21**	**.04-.39**	**.*019***	.04	-.13-.21	.*624*	.09	-.04-.22	.*169*	-.00	-.05-.05	.*966*	.01	-.05-.06	.*802*	.05	-.01-.11	.*079*
Concern	-.01	-.14-.12	.*840*	-.03	-.15-.10	.*696*	.03	-.07-.12	.*564*	.01	-.05-.08	.*709*	.02	-.04-.09	.*480*	.03	-.04-.11	.*398*
Expected return to everyday life	.04	-.11-.19	.*634*	-.02	-.17-.12	.*760*	.00	-.11-.11	.*974*	.02	-.05-.09	.*600*	.01	-.07-.09	.*805*	-.02	-.10-.07	.*725*
Informed	**.20**	**.08-.32**	**.*001***	**.16**	**.05-.27**	**.*006***	**.23**	**.15-.32**	***<* .*001***	.01	-.03-.06	.*612*	.02	-.03-.07	.*399*	-.01	-.06-.05	.*769*
Trust in COVID-19 measures	-.06	-.34-.22	.*667*	-.10	-.37-.17	.*464*	-.11	-.31-.09	.*267*	.06	-.02-.14	.*154*	.07	-.02-.16	.*138*	.02	-.08-.11	.*680*
SARS-CoV-2 infection^a^	-1.0	-3.75–1.75	.*475*	-1.25	-3.89–1.39	.*353*	.03	-1.95–2.0	.*977*	-.15	-1.71–1.40	.*848*	-.021	-1.89–1.47	.*810*	-.10	-1.91–1.71	.*915*
Blood donation frequency	**.88**	**.29–1.47**	**.*004***	**.81**	**.25–1.38**	**.*005***	**.86**	**.44–1.29**	***<* .*001***									
Time period fixed effects	Yes			Yes			Yes			Yes			Yes			Yes		
No. of respondents	60			60			60			152			152			152		
No. of observations	300			300			300			760			760			760		
R^2^	.195			.214			.319			.019			.031			.024		

Note: First-difference estimator. Significant results are marked in bold.

^a^ (1: yes / 0: no)

Results show that an increase in self-efficacy over time leads to an increase in donation intentions in the short term, medium term, and long term (Table 3, Model 1–3) for active donors. Likewise, positive changes in personal moral norms over time lead to an increase in donation intentions in the short, medium, and long term. Concerning perceived impact, an increase over time leads to an increase in short-term donation intentions but does not predict medium or long-term donation intentions. The results also show that changes in the frequency of blood donations of active donors increase donation intentions in the short, medium, and long term. Regarding the pandemic-specific variables, there is only one significant effect on donation intentions of active donors: An increase in donors feeling “informed” about blood donations during the pandemic over time predicts an increase in donation intentions in the short, medium, and long term. Changes in other pandemic-specific variables do not significantly predict donation intentions.

Concerning donation intentions of inactive donors, changes over time can solely be explained by two variables in our model resulting in a considerably lower R^2^ (see Table 3). First, an increase in self-efficacy over time leads to an increase in donation intentions in the short, medium, and long term. Second, an increase in personal moral norms over time predicts increases in donation intentions in the short, medium, and long term. Changes in the pandemic-specific variables do not significantly predict donation intentions of inactive donors.

To check the robustness of our findings, we also estimate an alternative variant of our panel model using unbalanced data (i.e., all participants who took part in our panel study; S32 Table in S1 File). Overall, our results are highly robust and confirm the direction of the effects as well as the statistical significance.

## Discussion

Our results demonstrate how a pandemic can decrease intentions to donate blood. We show this in the current setting of the COVID-19 pandemic relying on pre-pandemic and pandemic data. Prior literature states that individuals show higher willingness to help during disasters, including increased willingness to donate blood [2, 3]. For example, blood banks had to inform donors that there was no blood shortage after the terror attacks on September 11, yet thousands of donors went to donate [3]. Due to the significant differences of a pandemic compared to other crises, we propose that individuals behave differently, that is, are less willing to donate blood. A pandemic is a long-term crisis that affects and potentially poses a health threat to all individuals. We argue that this decrease in donation intention occurs due to a decrease in personal moral norms, self-efficacy, and perceived impact of a donation during a pandemic. Our results support hypothesis H1 demonstrating that blood donation behavior can be severely compromised, especially following pandemic outbreaks. Individuals report significantly lower donation intentions for the next six months, and even lower intentions for the long term compared to pre-pandemic donation intentions. Therefore, the pandemic could cause long-lasting negative effects on donation behavior.

Continued awareness of the need for blood and early retention strategies are crucial. Although recruiting new donors is also a possibility, retention of these has shown to be very challenging. Only 25–35% of first-time donors make a second donation [43]. Thus, it is of the utmost importance to understand the pandemic effect on blood donors’ donation intentions. Results in prior literature are not differentiated by donor status (i.e., active or inactive blood donors). Our results show that donation intentions of active donors are less affected by the pandemic in comparison to inactive donors. In fact, short-term blood donation intentions of active donors increase at the beginning of the pandemic compared to the pre-pandemic phase. This is partially in line with prior literature as prior studies do not differentiate between donation history or donor status. In addition, donation intentions of active donors do not change as the pandemic progresses. However, medium and long-term donation intentions of inactive donors decrease, undermining the increase in donation intentions of active donors. We also observe a negative COVID-19 effect for non-donors. Overall, this results in a negative pandemic effect on donation intentions. These results are alarming as the need for blood is crucially higher in the medium and long term when postponed surgeries resume [12–16].

Furthermore, we show that the underlying mechanisms leading to the negative pandemic effect also differ between active and inactive donors. First, inactive donors feel less able to donate during the pandemic compared to pre-pandemic, meaning their self-efficacy to donate blood decreases. On the contrary, active donors report a temporary higher value of self-efficacy at the beginning of the pandemic compared to the baseline level of self-efficacy from the pre-pandemic phase. This suggests active donors are in fact aware of their ability and eligibility to donate. The higher level of self-efficacy may reflect their increased desire to confirm that they are healthy. This finding matches prior research regarding donation behavior during crises. When faced with their own mortality, individuals are likely to seek to undermine their mortality by proving their health. However, over time (t_1_ compared to t_6_), this increase in self-efficacy significantly drops. Second, inactive donors expect their donation to have less impact during a pandemic in comparison to pre-pandemic. Faced with the ongoing crisis of the COVID-19 pandemic, their perceived individual contribution of a blood donation becomes smaller. However, active donors’ perceived donation impact is not altered by COVID-19. Third, in contrast to previous donor motivation literature, our results show that personal moral norms are not constant and can be affected by a pandemic. Recent literature on the effects of COVID-19 demonstrates how personal moral norms regarding prosocial behavior has changed during the pandemic [5]. We complement this research by showing that personal moral norms in the context of blood donations are altered and link this to blood donation intentions. Regardless of donor status, blood donors feel less responsible and less morally obliged to donate. As individuals are also required to follow legal restrictions (e.g., social distancing, contact restrictions, working from home, etc.) in order to reduce infection rates, their perceived responsibility to behave prosocially in other ways, that is, by donating blood, may be attenuated.

We can also show that positive changes in personal moral norms and self-efficacy during the pandemic can boost blood donation intentions of both active and inactive donors. This elucidates the importance of retention and reactivation strategies. In line with our hypothesis H2, donation intentions decrease in the pandemic as personal moral norms drop. Blood banks must focus their retention appeals on the urgent need for blood to help others in need by increasing perceived responsibility and moral obligation. While, in general, all individuals are affected by the pandemic, there is still a significant amount of people who need blood (e.g., every two seconds someone in the US needs blood [44]). In line with hypothesis H3, blood donors also feel less able and capable to donate blood, especially in the long term. Self-efficacy is measured linked to the factor of health, thus, calling for strategies to highlight a) the donor’s health and eligibility and b) that all measures are taken to ensure the donor’s safety during blood collection. Moreover, results show that active donors are more willing to donate blood if they feel sufficiently informed about the issue of blood donations during COVID-19. This highlights the importance of information strategies throughout the pandemic to retain active donors. Although our results show that donation intentions of inactive donors are significantly lower compared to active donors and significantly more affected by the pandemic, only appealing to active donors is not likely sufficient. It is important to note that our results also show the potential in addressing inactive donors who, contrary to new donors, have already overcome potential psychological barriers.

This work makes three central contributions to blood donation literature and research on donation behavior during crises. First, our results demonstrate that a pandemic (i.e., COVID-19), contrary to other crises, can significantly decreases the willingness to help, specifically with blood donation intentions. This negative impact also affects long-term blood donation intentions. Intention to donate blood does not increase but decreases when compared to pre-pandemic. We argue that a pandemic is different to other crises in the sense that it is a long-term large-scale crisis that affects all individuals and potentially threatens one’s own, and others’, health, thus, leading to different blood donation behavior. Second, we show that this decrease in donation intentions in the pandemic, compared to pre-pandemic, is due to a decrease in both personal moral norms and self-efficacy. We complement recent research [5] by showing that personal moral norms in the context of blood donations are affected by a pandemic. Inactive donors’ intentions to donate, in addition to a drop in personal moral norms and self-efficacy, are further decreased by a drop in perceived impact. Third, we distinguish by donation history (i.e., donor, non-donor) as well as donor status (i.e., active, inactive). This distinction is highly relevant as the size of the pandemic effect on donation intentions differs between individuals. Whereas inactive and non-donors report lower blood donation intentions in the pandemic compared to pre-pandemic, we find the opposite effect for active donors in the short term. This is in line with previous literature which states that situational factors (negatively) affect donations of individuals who are usually not committed to the cause more than individuals who are already committed to the cause [45–47]. Overall, these findings highlight the importance of retention and recruitment strategies during a pandemic, and offer evidence-based implications for blood banks.

### Limitations and conclusion

This research has some limitations. First, we measure blood donation intentions. Although donation intentions do not show actual donations, they make donation predictions in the medium and long term possible. In fact, prior literature has demonstrated that donation intentions are strong predictors of real donation behavior [25, 29]. Second, the underlying mechanisms that we considered in our analyses have a higher explanatory power for active than for inactive donors. Additional explanatory variables would be needed to better explain the donation intentions of inactive donors. It would also be insightful to further investigate the underlying mechanisms of non-donors as we do not measure personal moral norms, perceived impact, and self-efficacy for non-donors. Third, our data is limited by German context, capturing the effects of COVID-19 over a course of 12 weeks during the first pandemic phase (April to June 2020). However, at the time of our panel study, individuals may not have been able to fully assess the scale or longevity of the pandemic. Moreover, the end of the pandemic is still unknown.

Given that approximately 28.4 million planned surgeries (72.3%) worldwide have been postponed due to COVID-19 [17], it will take a long time to catch up. This means a higher level of blood supply will be required in the long term. Our results suggest that the intentions to donate blood during COVID-19 do not match the blood donations that will be needed, especially when surgeries are resumed. The end of the current pandemic is still unknown and our results show a significant decrease in perceived personal responsibility and moral obligation (i.e., personal moral norms) regarding blood donations, and decreasing self-efficacy as the pandemic progresses, leading to a downward spiral. This is alarming because the more time passes, the less likely a donor is to return. Blood banks must emphasize the importance of solidarity in times of a pandemic more than ever to effectively trigger people’s prosocial behavior.

## Supporting information

S1 File (PDF)
