# Supplementary material for: The impact of COVID-19 on blood donations
Source: PLoS One. 2022 Mar 24;17(3):e0265171. doi: 10.1371/journal.pone.0265171 (PMC8946670; doi:10.1371/journal.pone.0265171)
Supplement: S1 File — (PDF) [file pone.0265171.s001.pdf]

Supporting Information for

The impact of COVID-19 on blood donations

Besarta Veseli, Sabrina Sandner, Sinika Studte, & Michel Clement

Correspondence to: michel.clement@uni-hamburg.de

This file includes:

Supplementary information text

Figures S1 – S2

Tables S1 – S32

Supplementary Information Text

Materials. This paper is based on an online study conducted in April 2019 (n = 2,449) and a biweekly six-wave panel study carried out from April to June 2020 (n_1 = 1,499, n_2 = 1,189, n_3 = 1,057, n_4 = 998, n_5 = 936, n_6 = 818). All studies were programmed using *Unipark* survey software. The participants were approached by *respondi*, a commercial panel provider. The respondents in our panel are between 18 and 75 years old and represent the age and gender of the population. The percentage of blood donors in our sample is unusually high compared to the eligible population (see Table S1), but the respondents were not invited according to this criterion, and it is not uncommon for blood donors to be rather overrepresented among survey respondents. The respondents were paid with so-called mingle points, which could then be redeemed for cash or shopping vouchers. Participants received mingle points worth one Euro for 20 minutes of questioning.

Methods.

Moderated Mediation Analysis. We conducted a moderated mediation analysis using model 7 of the PROCESS macro (all results can be found in Tables S14-S31, the conceptual framework is shown in Figure S1). We analyze the mediating role (indirect effects) of personal moral norms, perceived impact, and self-efficacy. We also investigate the moderating role (interaction effect) of donor status to account for effects based on donation recency (active vs. inactive donors). We classify blood donors as inactive if they have not donated in the last 24 months, following Red Cross classifications [38].

Pre-Pandemic vs. Pandemic in the Short Term. The interaction effect between the pandemic effect and donor status is positive and significant on personal moral norms (b=.537, se=.153, p=.001), perceived impact (b=1.019, se=1.47, p<.001), and self-efficacy (b=.733, se=.134, p<.001). The pandemic effect on these reported donor motivations is therefore less negative for active donors. Firstly, COVID-19 significantly reduces personal moral norms (b=-1.013, se= .096, p<.001), perceived impact (b=-.960, se= .092, p<.001), and self-efficacy (b=-.505, se= .084, p<.001), which conversely significantly drive donation intentions. The direct effect of the pandemic on donation intention is not significant. All detailed results of the short term are shown in Table S14.

Next, we analyze the indirect effects via proposed donor motivations between pre-pandemic and pandemic (t_1). The indirect effect of the pandemic on donation intentions through personal moral

norms, based on bootstrapping with 10,000 replications, is significant and negative for both active (95% b=-.208, CI: -.309 to -.109) and inactive donors (95% b=-.442, CI: -.557 to -.334). Hence, as the positive interaction effect indicates, the indirect effect is less negative for active donors. Although the indirect effect via perceived impact is not significant for active donors (b=.010, CI: -.015 to .036), it is significant and negative for inactive donors (95% b=-.155, CI: -.224 to -.092). Lastly, the indirect effect via self-efficacy is significant for both active and inactive donors. However, the indirect effect is positive for active donors (95% b=.115, CI: .050 to .181) and negative for inactive donors (95% b=-.254, CI: -.363 to .152). In conclusion, the pandemic effect on donation intention (t_1) is mediated by personal moral norms (PMN), perceived impact (PI), and self-efficacy (SE) and is moderated by donor status. Figure S2 also displays the indirect effects of active and inactive donors (t_2 to t_6). The negative indirect effect via personal moral norms decreases.
All detailed results of the indirect effects between pre-pandemic and pandemic (t_2 to t_6) are shown in Tables S17, S20, S23, S26, and S29.

Pre-Pandemic vs. Pandemic in the Medium Term. The interaction effect between the pandemic effect and donor status is positive and significant on personal moral norms (b=.537, se=.153, p=.001), perceived impact (b=1.019, se=1.47, p<.001), and self-efficacy (b=.733, se=.134, p<.001). The pandemic effect on these reported donor motivations is therefore less negative for active donors. Firstly, COVID-19 significantly reduces personal moral norms (b=-1.013, se= .096, p<.001), perceived impact (b=-.960, se= .092, p<.001), and self-efficacy (b=-.505, se= .084, p<.001), which conversely significantly drive donation intention. The direct effect of the pandemic on donation intention is not significant. All detailed results for the medium term are shown in Table S15.

Next, we analyze the indirect effects via the proposed donor motivations between pre-pandemic and pandemic (t_1). The indirect effect of the pandemic on donation intentions through personal moral norms, based on bootstrapping with 10,000 replications, is significant and negative for both active (95% b=-.181, CI: -.273 to -.094) and inactive donors (95% b=-.386, CI: -.492 to -.285). Hence, as the positive interaction effect indicates, the indirect effect is less negative for active donors. Although the indirect effect via perceived impact is not significant for active donors (b=.012, CI: -.020 to .045), it is significant and negative for inactive donors (95% b=-.201, CI: -.280 to -.129). Lastly, the indirect effect via self-efficacy is significant for both active and inactive donors. However, the indirect effect is positive for active donors (95%b=.137, CI: .059

to .215) and negative for inactive donors (95% b=-.303, CI: -.430 to -.182). In conclusion, the pandemic effect on donation intention (t_1) is mediated by personal moral norms (PMN), perceived impact (PI), and self-efficacy (SE) and is moderated by donor status. Figure S2 also displays the indirect effects of active and inactive donors (t_2 to t_6). The negative indirect effect via personal moral norms decreases.

All detailed results of the indirect effects between pre-pandemic and pandemic (t_2 to t_6) are shown in Tables S18, S21, S24, S27, and S30.

Pre-Pandemic vs. Pandemic in the Long Term. The interaction effect between the pandemic effect and donor status is positive and significant on personal moral norms (b=.537, se=.153, p=.001), perceived impact (b=1.019, se=1.47, p<.001), and self-efficacy (b=.733, se=.134, p<.001). The pandemic effect on these reported donor motivations is therefore less negative for active donors. Firstly, COVID-19 significantly reduces personal moral norms (b=-1.013, se= .096, p<.001), perceived impact (b=-.960, se= .092, p<.001), and self-efficacy (b=-.505, se= .084, p<.001), which conversely significantly drive donation intention. The direct effect of the pandemic on donation intention is negative (-.350, p<.001).

We analyze the indirect effects between pre-pandemic and pandemic (t_1). All detailed results in the long term are shown in Table S16. The indirect effect of the pandemic on donation intentions through personal moral norms, based on bootstrapping with 10,000 replications, is significant and negative for both active (95% b=-.140, CI: -.215 to -.072) and inactive donors (95% b=-.297, CI: -.390 to -.210). Although the indirect effect via perceived impact is not significant for active donors (95% b=.015, CI: -.024 to .055), it is significant and negative for inactive donors (95% b=-.246, CI: -.335 to -.165). The indirect effect via self-efficacy is significant and positive for active donors (95% b=.145, CI: .063 to .228), and significant and negative for inactive donors (95% b=-.320, CI: -.453 to -.192).

All detailed results of the indirect effects between pre-pandemic and pandemic (t_2 to t_6) are shown in Tables S19, S22, S25, S28, and S31.

Panel Analysis. In our panel study analysis, we account for panel mortality by relying on balanced data in our panel model, i.e., we only include respondents who completed all six waves of the study in the analysis (i.e., 593 respondents, Table S2). To measure the effect of changes in the underlying mechanisms on donation intention over time, we used a fixed effects panel estimator with a first-difference approach to eliminate individual effects. This approach controls

for unobservable fixed effects as well as for unobservable (time-invariant) differences between individuals [41]. Although we control for age and gender, the respective effects are not displayed as these are time-fixed effects. Detailed statistical results and the correlation matrices are provided in Tables S4-S7.

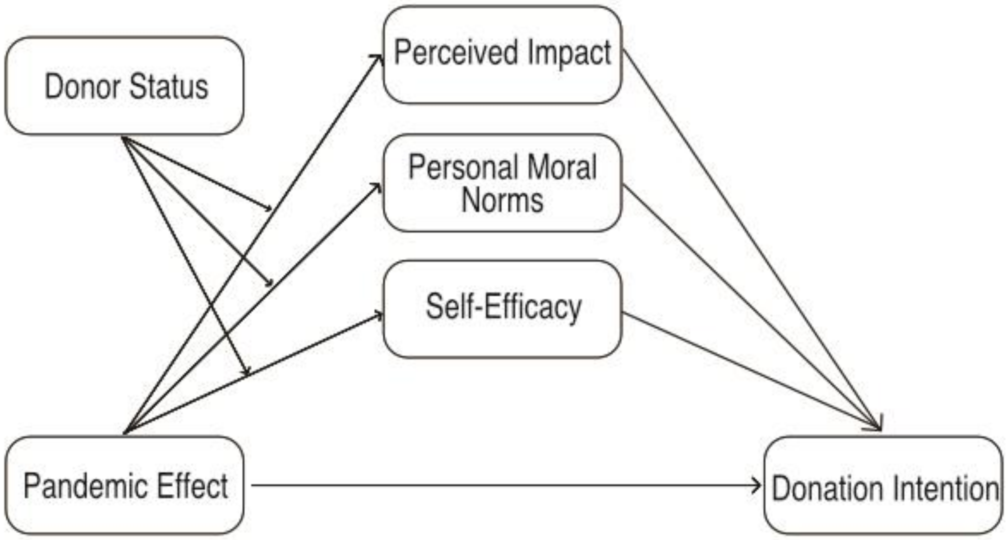

Figure S1. Moderated mediation.

Donor status serves as moderator, whereby 0 = inactive donor and 1 = active donor. Perceived impact, personal moral norms, and self-efficacy serve as mediators.

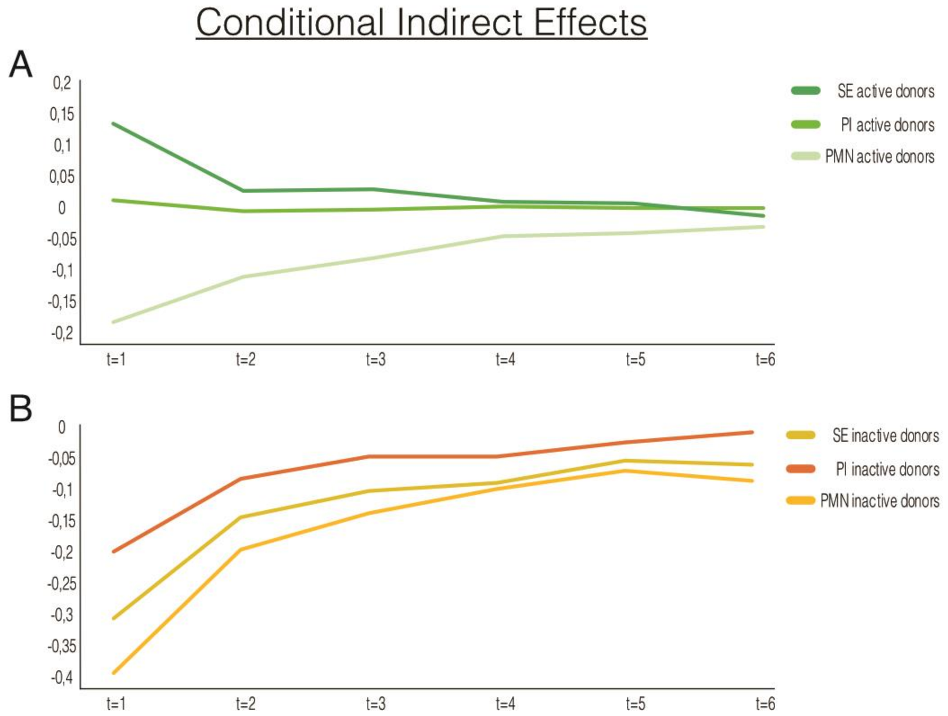

Figure S2. Conditional indirect effects.

Graphical representation of the conditional indirect effects according to the moderated mediation analysis for pandemic (t_1 to t_6) with pre-pandemic serving as baseline. Results are divided into active (A) and inactive (B) donors.

	Pre-Pandemic (unweighted) (N=2,449)		Pandemic (*t*= *1*, unweighted) (N=1,499)		Pandemic (*t*= *1*, weighted) (N=1,490)	
	Absolute Number	Percentage	Absolute Number	Percentage	Absolute Number	Percentage
**Gender**						
Female	1,086	44.3	749	50.0	665	44.6
Male	1,349	55.1	750	50.0	826	55.4
Diverse	14	.6	0	0	0	0
**Average Age (in years)**	48.29		47.06		45.96	
**Donation History**						
Blood donor	1,280	52.3	684	45.6	781	52.4
Non-donor	1,169	47.7	815	54.4	709	47.6
**Donor Status**						
Active donors	640	50.0	233	34.1	391	50.0
Inactive donors	640	50.0	451	65.9	391	50.0

Table S1. Descriptives pre-pandemic and pandemic (*t=1*)

Descriptives for the pre-pandemic and pandemic (*t=1)*, including unweighted and weighted values. We weighted the data for gender, donation history, and donor status.

	Pandemic (*t*= *1*, unbalanced) (N=1,499)		Pandemic (*t*= *1*, balanced) (N=593)	
	Absolute Number	Percentage	Absolute Number	Percentage
**Gender**				
Female	749	50.0	282	47.55
Male	750	50.0	311	52.45
Diverse	0	0	0	0
**Average Age (in years)**	47.06		51.14	
**Donation History**				
Blood donor	684	45.6	212	35.75
Non-donor	815	54.4	381	64.25
**Donor Status**				
Active donors	233	34.1	60	28.30
Inactive donors	451	65.9	152	71.70

Table S2. Descriptives for the pandemic, unbalanced and balanced (*t=1*)

Descriptives for the pandemic (*t=1)*. Balanced includes only those participants who completed all six waves.

	ACTIVE DONORS				INACTIVE DONORS			
	Pre-Pandemic		Pandemic (t_1)		Pre-Pandemic		Pandemic (t_1)	
	α	M (SD)	α	M (SD)	α	M (SD)	α	M (SD)
**Intention *[26]***								
I intend to donate blood on the next possible date.		5.36 (1.72)		5.72 (1.76)		2.38 (1.71)		2.00 (1.64)
I intend to donate blood over the next six months.		5.99 (1.41)		6.12 (1.51)		2.78 (1.96)		2.20 (1.86)
It is likely that I will donate blood in the future.		6.37 (1.15)		6.42 (1.28)		3.67 (2.29)		2.65 (2.14)
**Personal Moral Norms [26]**	.808	5.08 (1.31)	.839	4.65 (1.58)	.869	4.05 (1.65)	.843	3.04 (1.73)
I feel a personal responsibility to give blood.								
I feel a moral obligation to give blood.								
I feel a social obligation to give blood.								
Sometimes I feel guilty that I do not donate blood.								
**Perceived Impact *[37]***	.702	5.99 (1.02)	.775	6.06 (1.01)	.850	5.31 (1.59)	.924	4.35 (2.05)
My blood donation is needed.								
My blood donation makes a difference.								
My blood donation has an impact.								
**Self-Efficacy *[27]***	.661	6.21 (0.93)	.685	6.45 (0.79)	.634	5.17 (1.51)	.688	4.66 (1.82)
If I wanted to, I would be able to give blood as long as my health allows it.								
I think myself capable of continuing to give blood as long as my health allows it.								
I find it hard to give blood time after time.								

Table S3. Mean values and standard deviations of *active* and *inactive donors.*

Mean values and standard deviations of *active* and *inactive donors* regarding donation intentions, personal moral norms, perceived impact, and self-efficacy for pre-pandemic as well as for pandemic (*t=1)*. Cases are weighted for gender, donation history, and donor status.

ACTIVE DONORS	t=1		t=2		t=3		t=4		t=5		t=6	
	M (SD)	Min - Max	M (SD)	Min - Max	M (SD)	Min - Max	M (SD)	Min - Max	M (SD)	Min - Max	M (SD)	Min - Max
**Intention *[26]***												
I intend to donate blood on the next possible date.	5.67 (1.78)	1 - 7	5.37 (1.89)	1 - 7	5.67 (1.65)	1 - 7	5.33 (2.03)	1 - 7	5.51 (1.92)	1 -7	5.54 (1.94)	1 - 7
I intend to donate blood over the next six months.	6.11 (1.49)	1 - 7	5.85 (1.78)	1 – 7	5.89 (1.61)	1 – 7	5.71 (1.80)	1 – 7	5.83 (1.73)	1 – 7	5.75 (1.95)	1 - 7
It is likely that I will donate blood in the future.	6.40 (1.28)	1 - 7	6.26 (1.48)	1 – 7	6.21 (1.36)	1 – 7	6.28 (1.44)	1 – 7	6.16 (1.51)	1 – 7	6.21 (1.49)	1 - 7
**Personal Moral Norms *[26]***	4.60 (1.60)	1 – 7	4.53 (1.66)	1 – 7	`4.45 (1.81)	1 – 7	4.62 (1.73)	1 – 7	4.57 (1.76)	1 – 7	4.69 (1.77)	1 - 7
**Perceived Impact *[37]***	6.06 (1.00)	1 – 7	5.97 (1.12)	1 – 7	5.98 (1.12)	1 – 7	6.08 (1.10)	1 – 7	6.03 (1.24)	1 – 7	6.03 (1.12)	1 - 7
**Self-Efficacy *[27]***	6.44 (.81)	1 – 7	6.31 (.95)	1 – 7	6.36 (.96)	1 – 7	6.28 (.87)	1 – 7	6.29 (.90)	1 – 7	6.09 (1.04)	1 - 7
**Concern**	3.27 (1.67)	1 – 7	3.16 (1.61)	1 – 7	3.01 (1.56)	1 – 7	2.99 (1.53)	1 – 7	2.92 (1.66)	1 – 7	2.86 (1.68)	1 – 7
**Expected Return to Everyday Life**	3.74 (1.29)	1 – 7	4.39 (1.38)	1 – 7	4.61 (1.41)	1 – 7	4.66 (1.36)	1 – 7	4.49 (1.42)	1 – 7	4.40 (1.47)	1 – 7
**Informed**	4.97 (2.03)	1 – 7	5.25 (1.90)	1 – 7	5.47 (1.81)	1 – 7	5.38 (1.86)	1 – 7	5.55 (1.70)	1 – 7	5.75 (1.54)	1 – 7
**Trust in COVID-19 Measures at Blood Collection**	6.19 (.99)	1 – 7	6.18 (.96)	1 – 7	6.25 (.93)	1 – 7	6.29 (.97)	1 – 7	6.16 (1.01)	1 – 7	6.21 (1.01)	1 – 7
**SARS-CoV-2 Infection** *(% of respondents infected)*	0%	0 – 1	1.11%	0 – 1	0.66%	0 – 1	0.77%	0 – 1	1.67%	0 – 1	1.96%	0 – 1

Table S4. Descriptive statistics of model variables for the *active donors.*

Mean values and standard deviations of the *active donors* regarding donation intentions, personal moral norms, perceived impact, and self-efficacy for pandemic (*t=1* to *t=6)*.

INACTIVE DONORS	t=1		t=2		t=3		t=4		t=5		t=6	
	M (SD)	Min - Max	M (SD)	Min - Max	M (SD)	Min - Max	M (SD)	Min - Max	M (SD)	Min - Max	M (SD)	Min - Max
**Intention *[26]***												
I intend to donate blood on the next possible date.	2.00 (1.63)	1 - 7	1.86 (1.56)	1 - 7	1.77 (1.50)	1 - 7	1.66 (1.32)	1 - 7	1.81 (1.55)	1 -7	1.61 (1.33)	1 - 7
I intend to donate blood over the next six months.	2.19 (1.85)	1 - 7	2.01 (1.72)	1 – 7	1.85 (1.61)	1 – 7	1.77 (1.48)	1 – 7	1.86 (1.59)	1 – 7	1.62 (1.32)	1 - 7
It is likely that I will donate blood in the future.	2.65 (2.13)	1 - 7	2.48 (2.03)	1 – 7	2.24 (1.97)	1 – 7	2.10 (1.83)	1 – 7	2.26 (1.92)	1 – 7	2.05 (1.78)	1 - 7
**Personal Moral Norms *[26]***	3.04 (1.72)	1 – 7	3.03 (1.78)	1 – 7	2.91 (1.79)	1 – 7	2.92 (1.81)	1 – 7	3.05 (1.84)	1 – 7	2.66 (1.75)	1 - 7
**Perceived Impact *[37]***	4.35 (2.04)	1 – 7	4.34 (2.06)	1 – 7	4-29 (2.10)	1 – 7	4.21 (2.07)	1 – 7	4.35 (2.10)	1 – 7	4.34 (2.15)	1 - 7
**Self-Efficacy *[27]***	4.66 (1.82)	1 – 7	4.64 (1.77)	1 – 7	4.61 (1.81)	1 – 7	4.48 (1.82)	1 – 7	4.62 (1.77)	1 – 7	4.45 (1.75)	1 - 7
**Concern**	3.57 (1.64)	1 – 7	3.17 (1.60)	1 – 7	3.23 (1.73)	1 – 7	3.13 (1.65)	1 – 7	3.12 (1.60)	1 – 7	2.99 (1.66)	1 – 7
**Expected Return to Everyday Life**	3.69 (1.35)	1 – 7	4.31 (1.43)	1 – 7	4.63 (1.49)	1 – 7	4.66 (1.49)	1 – 7	4.77 (1.51)	1 – 7	4.88 (1.59)	1 – 7
**Informed**	3.80 (2.27)	1 – 7	4.12 (2.29)	1 – 7	4.14 (2.27)	1 – 7	4.38 (2.14)	1 – 7	4.50 (2.16)	1 – 7	4.42 (2.21)	1 – 7
**Trust in COVID-19 Measures at Blood Collection**	5.67 (1.26)	1 – 7	5.65 (1.34)	1 – 7	5.78 (1.24)	1 – 7	5.77 (1.19)	1 – 7	5.83 (1.22)	1 – 7	5.84 (1.23)	1 – 7
**SARS-CoV-2 Infection** *(% of respondents infected)*	0.44%	0 – 1	0.28%	0 – 1	0%	0 – 1	0.34%	0 – 1	0.37%	0 – 1	0%	0 – 1

Table S5. Descriptive statistics of model variables for the *inactive donors.*

Mean values and standard deviations of the *inactive donors* regarding donation intentions, personal moral norms, perceived impact, and self-efficacy for pandemic (*t=1* to *t=6*).

ACTIVE DONORS	Self-efficacy	Personal moral norms	Perceived impact	Concern	Expected return to everyday life	Informed	Trust in COVID-19 measures	SARS-CoV-2 infection	Blood donation frequency
Self-efficacy	1.000								
Personal moral norms	0.056	1.000							
Perceived impact	0.565	0.450	1.000						
Concern	-0.234	-0.039	-0.246	1.000					
Expected return to everyday life	-0.004	0.048	0.035	0.228	1.000				
Informed	0.111	0.167	0.283	-0.228	-0.159	1.000			
Trust in COVID-19 measures	0.089	0.282	0.311	-0.372	-0.055	0.297	1.000		
SARS-CoV-2 infection	0.022	-0.095	0.015	-0.064	-0.008	0.014	0.010	1.000	
Blood donation frequency	0.278	0.044	0.141	0.136	-0.041	0.037	-0.044	0.023	1.000

Table S6. Correlation matrix for *active donors*.

Correlation matrix includes all model variables of the panel analysis.

**INACTIVE DONORS**	Self-efficacy	Personal moral norms	Perceived impact	Concern	Expected return to everyday life	Informed	Trust in COVID-19 measures	SARS-CoV-2 infection	Blood donation frequency
Self-efficacy	1.000								
Personal moral norms	0.245	1.000							
Perceived impact	0.359	0.380	1.000						
Concern	-0.095	0.012	-0.026	1.000					
Expected return to everyday life	-0.012	-0.043	0.045	0.025	1.000				
Informed	-0.111	0.103	0.039	-0.096	0.119	1.000			
Trust in COVID-19 measures	0.049	0.170	0.159	-0.070	0.027	0.263	1.000		
SARS-CoV-2 infection	-0.074	-0.051	0.009	-0.037	0.041	0.057	0.008	1.000	
Blood donation frequency	0.070	0.022	0.038	0.013	0.044	-0.011	-0.034	-0.032	1.000

Table S7. Correlation matrix for *inactive donors*.

Correlation matrix includes all model variables of the panel analysis.

	$M(SD)_{2019}$	$M(SD)_{t1}$	*p*
**Blood donors**			
Short-term donation intentions	3.87(2.27)	3.86(.252)	*0.953*
Medium-term donation intentions	4.39(2.34)	4.16(2.59)	*0.039*
Long-term donation intentions	5.02(2.26)	4.54(2.58)	*<0.001*
**Active donors**			
Short-term donation intentions	5.36(1.72)	5.72(1.76)	*0.001*
Medium-term donation intentions	5.99(1.41)	6.12(1.51)	*0.180*
Long-term donation intentions	6.37(1.15)	6.42(1.28)	*0.452*
**Inactive donors**			
Short-term donation intentions	2.38(1.71)	2.00(1.64)	*<0.001*
Medium-term donation intentions	2.78(1.96)	2.20(1.86)	*<0.001*
Long-term donation intentions	3.67(2.29)	2.65(2.14)	*<0.001*
**Non-donors**			
Short-term donation intentions	*N/A*	*N/A*	*N/A*
Medium-term donation intentions	2.30(1.62)	1.62(1.34)	*<0.001*
Long-term donation intentions	2.88(2.00)	2.09(1.76)	*<0.001*

Table S8. T-Test results of blood donation intentions for pre-pandemic (2019) and pandemic (t_1).
We weighted the cases for gender, donation history, and donor status.

	M(SD)$_{2019}$	M(SD)$_{t1}$	*p*
**Blood donors**			
Personal moral norms	4.56(1.58)	3.85(1.84)	*<0.001*
Perceived impact	5.65(1.38)	5.21(1.82)	*<0.001*
Self-efficacy	5.69(1.36)	5.56(1.66)	*0.053*
**Active donors**			
Personal moral norms	5.08(1.31)	4.65(1.58)	*<0.001*
Perceived impact	5.99(1.02)	6.06(1.01)	*0.363*
Self-efficacy	6.21(0.93)	6.45(0.79)	*<0.001*
**Inactive donors**			
Personal moral norms	4.05(1.65)	3.04(1.73)	*<0.001*
Perceived impact	5.31(1.59)	4.35(2.05)	*<0.001*
Self-efficacy	5.17(1.51)	4.66(1.82)	*<0.001*

Table S9. T-Test results of underlying mechanisms (personal moral norms, perceived impact, and self-efficacy) for pre-pandemic (2019) and pandemic (t_1). We weighted the cases for gender, donation history, and donor status.

	DFn	DFd	F	p_{adj}
**Blood donors**				
Short-term donation intentions	4.56	962.15	1.687	*0.284*
Medium-term donation intentions	4.58	967.16	2.061	*0.148*
Long-term donation intentions	4.24	893.59	3.251	*<0.001*
**Active donors**				
Short-term donation intentions	4.07	195.20	1.057	*0.760*
Medium-term donation intentions	3.52	168.75	0.613	*1.000*
Long-term donation intentions	3.46	166.30	0.874	*0.936*
**Inactive donors**				
Short-term donation intentions	3.73	499.21	0.719	*1.000*
Medium-term donation intentions	3.86	517.83	1.569	*0.366*
Long-term donation intentions	4.22	566.07	3.428	*0.016*
**Non-donors**				
Short-term donation intentions	4.03	1461.77	3.424	*0.016*
Medium-term donation intentions	4.13	1499.03	2.501	*0.078*
Long-term donation intentions	4.27	1550.70	11.752	*<0.001*

Table S10. ANOVA (with repeated measures) results of donation intentions within the pandemic (t_1 to t_6).
p-values are adjusted using the Bonferroni multiple testing correction method.

ACTIVE VS INACTIVE DONORS															
	Short-term donation intentions					Medium-term donation intentions					Long-term donation intentions				
	$M(SD)_{Active}$	$M(SD)_{Inactive}$	Diff	[Low. - Up.]	*p*	$M(SD)_{Active}$	$M(SD)_{Inactive}$	Diff	[Low. - Up.]	*p*	$M(SD)_{Active}$	$M(SD)_{Inactive}$	Diff	[Low. - Up.]	*p*
t_1	5.67(1.78)	2.00(1.63)	3.678	3.247-4.110	<*.001*	6.11(1.49)	2.19(1.85)	3.914	3.475-4.353	<*.001*	6.40(1.28)	2.65(2.13)	3.754	3.276-4.232	<*.001*
t_2	5.37(1.89)	1.86(1.56)	3.515	3.025-4.005	<*.001*	5.85(1.78)	2.01(1.72)	3.844	3.345-4.343	<*.001*	6.26(1.48)	2.48(2.03)	3.782	3.239-4.326	<*.001*
t_3	5.64(1.68)	1.78(1.50)	3.859	3.327-4.392	<*.001*	5.85(1.65)	1.85(1.61)	4.006	3.464-4.547	<*.001*	6.18(1.42)	2.24(1.98)	3.939	3.349-4.529	<*.001*
t_4	5.33(2.03)	1.66(1.32)	3.671	3.109-4.233	<*.001*	5.72(1.80)	1.77(1.48)	3.944	3.372-4.517	<*.001*	6.28(1.44)	2.19(1.83)	4.176	3.553-4.799	<*.001*
t_5	5.48(1.95)	1.81(1.56)	3.666	3.079-4.253	<*.001*	5.79(1.78)	1.87(1.59)	3.926	3.328-4.525	<*.001*	6.12(1.57)	2.26(1.92)	3.854	3.203-4.506	<*.001*
t_6	5.50(1.98)	1.61(1.34)	3.891	3.253-4.529	<*.001*	5.71(2.00)	1.62(1.32)	4.084	3.434-4.733	<*.001*	6.22(1.48)	2.03(1.75)	4.185	3.477-4.892	<*.001*

Table S11. ANOVA results of donation intentions of *active and inactive donors* within the pandemic (t_1 to t_6).

We compare donation intentions of *active* and *inactive donors* within the pandemic. Used post-hoc test: Tukey HSD.

	DFn	DFd	F	p_{adj}
**Blood donors**				
Personal moral norms	4.68	988.44	0.506	*0.760*
Perceived impact	4.00	844.93	0.548	*0.700*
Self-efficacy	4.77	1006.00	1.163	*0.326*
**Active donors**				
Personal moral norms	4.08	195.67	0.761	*1.000*
Perceived impact	4.11	197.07	0.673	*1.000*
Self-efficacy	5.00	240.00	2.782	*0.036*
**Inactive donors**				
Personal moral norms	4.65	623.22	0.547	*1.000*
Perceived impact	4.05	542.95	0.751	*1.000*
Self-efficacy	5.00	670.00	0.483	*1.000*

Table S12. ANOVA (with repeated measures) results of the underlying mechanisms within the pandemic (t_1 to t_6).
Used post-hoc test: Bonferroni.

ACTIVE VS INACTIVE DONORS															
	Personal moral norms					Perceived impact					Self-efficacy				
	$M(SD)_{Active}$	$M(SD)_{Inactive}$	Diff	[Lower - Upper]	*p*	$M(SD)_{Active}$	$M(SD)_{Inactive}$	Diff	[Lower - Upper]	*p*	$M(SD)_{Active}$	$M(SD)_{Inactive}$	Diff	[Lower - Upper]	*p*
t_1	4.60(1.60)	3.04(1.72)	1.565	1.102-2.029	<*.001*	6.06(1.00)	4.35(2.04)	1.713	1.233-2.194	<*.001*	6.44(0.82)	4.66(1.82)	1.779	1.367-2.191	<*.001*
t_2	4.53(1.66)	3.03(1.78)	1.501	0.975-2.028	<*.001*	5.97(1.12)	4.34(2.06)	1.633	1.087-2.180	<*.001*	6.31(0.95)	4.64(1.77)	1.665	1.197-2.134	<*.001*
t_3	4.47(1.82)	2.89(1.78)	1.571	0.999-2.143	<*.001*	5.99(1.12)	4.28(2.10)	1.714	1.120-2.307	<*.001*	6.34(0.99)	4.61(1.81)	1.720	1.212-2.229	<*.001*
t_4	4.61(1.73)	2.92(1.81)	1.700	1.095-2.304	<*.001*	6.08(1.10)	4.21(2.07)	1.872	1.246-2.499	<*.001*	6.28(0.87)	4.48(1.82)	1.802	1.265-2.339	<*.001*
t_5	4.54(1.78)	3.08(1.84)	1.487	0.855-2.118	<*.001*	6.04(1.24)	4.34(2.10)	1.699	1.044-2.354	<*.001*	6.29(0.89)	4.62(1.76)	1.676	1.115-2.238	<*.001*
t_6	4.65(1.79)	2.67(1.75)	1.983	1.297-2.669	<*.001*	6.04(1.12)	4.32(2.15)	1.715	1.003-2.426	<*.001*	6.09(1.04)	4.44(1.75)	1.652	1.042-2.262	<*.001*

Table S13. ANOVA results of underlying mechanisms (personal moral norms, perceived impact, and self-efficacy) of *active and inactive donors* within the pandemic (t_1 to t_6).

We compare personal moral norms, perceived impact, and self-efficacy of active and inactive donors within the pandemic. Used post-hoc test: Tukey HSD.

	Personal Moral Norms				Perceived Impact				Self-Efficacy				Donation Intention[2]			
	b (se)	*p*	2.5%	97.5%	b (se)	*p*	2.5%	97.5%	b (se)	*p*	2.5%	97.5%	b (se)	*p*	2.5%	97.5%
Pandemic Effect	-1.013 (.096)	.000	-1.201	-.824	-.960 (.092)	.000	-1.141	-.780	-.505 (.084)	.000	-.670	-.341	.144 (.091)	.115	-.035	.323
Donor Status (DS)	1.029 (.087)	.000	.858	1.200	.694 (.084)	.000	.530	.859	1.046 (.076)	.000	.896	1.195				
Pandemic Effect × DS	.537 (.153)	.001	.236	.837	1.019 (.147)	.000	.731	1.308	.733 (.134)	.000	.471	.996				
Personal Moral Norms (PMN)													.437 (.030)	.000	.380	.495
Perceived Impact (PI)													.162 (.031)	.000	.100	.224
Self-Efficacy (SE)													.503 (.032)	.000	.441	.565
Constant	4.048 (.062)	.000	3.927	4.169	5.305 (.059)	.000	5.189	5.421	5.165 (.054)	.000	5.059	5.271	-1.903 (.189)	.000	-2.273	-1.532
Conditional Indirect Effect via PMN																
DS[1] (0)													-.442 (.057)		-.557	-.334
DS (1)													-.208 (.051)		-.309	-.109
Conditional Indirect Effect via PI																
DS (0)													-.155 (.034)		-.224	-.092
DS (1)													.010 (.013)		-.015	.036
Conditional Indirect Effect via SE																
DS (0)													-.254 (.054)		-.363	-.152
DS (1)													.115 (.034)		.050	.181
R^2	.195				.160				.201				.377			
df	3.000				3.000				3.000				4.000			
F (*p*)	158.059	.000			124.629	.000			163.933	.000			296.927	.000		

[1]0= Inactive donors, 1=active donors
[2] “I intend to donate blood on the next possible date.”

Table S14. Detailed results of the moderated mediation analysis on donation intentions in the *short term* regarding pre-pandemic and pandemic (*t=1*).

	Personal Moral Norms				Perceived Impact				Self-Efficacy				Donation Intention[2]			
	b (se)	*p*	2.5%	97.5%	b (se)	*p*	2.5%	97.5%	b (se)	*p*	2.5%	97.5%	b (se)	*p*	2.5%	97.5%
Pandemic Effect	-1.013 (.096)	.000	-1.201	-.824	-.960 (.092)	.000	-1.141	-.780	-.505 (.084)	.000	-.670	-.341	-.077 (.093)	.409	-.260	.106
Donor Status (DS)	1.029 (.087)	.000	.858	1.200	.694 (.084)	.000	.530	.859	1.046 (.076)	.000	.896	1.195				
Pandemic Effect × DS	.537 (.153)	.001	.236	.837	1.019 (.147)	.000	.731	1.308	.733 (.134)	.000	.471	.996				
Personal Moral Norms (PMN)													.381 (.030)	.000	.322	.440
Perceived Impact (PI)													.210 (.032)	.000	.147	.272
Self-Efficacy (SE)													.601 (.032)	.000	.538	.664
Constant	4.048 (.062)	.000	3.927	4.169	5.305 (.059)	.000	5.189	5.421	5.165 (.054)	.000	5.059	5.271	-1.952 (.193)	.000	-2.330	-1.574
Conditional Indirect Effect via PMN																
DS[1] (0)													-.386 (.052)		-.492	-.285
DS (1)													-.181 (.045)		-.273	-.094
Conditional Indirect Effect via PI																
DS (0)													-.201 (.039)		-.280	-.129
DS (1)													.012 (.017)		-.020	.045
Conditional Indirect Effect via SE																
DS (0)													-.303 (.064)		-.430	-.182
DS (1)													.137 (.040)		.059	.215
R^2	.195				.160				.201				.408			
df	3.000				3.000				3.000				4.000			
F (*p*)	158.059	.000			124.629	.000			163.933	.000			337.633	.000		

[1]0= Inactive donors, 1=active donors
[2] "I intend to donate blood over the next six months."

Table S15. Detailed results of the moderated mediation analysis on donation intentions in the *medium term* regarding pre-pandemic and pandemic (*t=1)*.

	Personal Moral Norms				Perceived Impact				Self-Efficacy				Donation Intention[2]			
	b (se)	*p*	2.5%	97.5%	b (se)	*p*	2.5%	97.5%	b (se)	*p*	2.5%	97.5%	b (se)	*p*	2.5%	97.5%
Pandemic Effect	-1.013 (.096)	.000	-1.201	-.824	-.960 (.092)	.000	-1.141	-.780	-.505 (.084)	.000	-.670	-.341	-.350 (.092)	.000	-.530	-.171
Donor Status (DS)	1.029 (.087)	.000	.858	1.200	.694 (.084)	.000	.530	.859	1.046 (.076)	.000	.896	1.195				
Pandemic Effect × DS	.537 (.153)	.001	.236	.837	1.019 (.147)	.000	.731	1.308	.733 (.134)	.000	.471	.996				
Personal Moral Norms (PMN)													.293 (.030)	.000	.235	.352
Perceived Impact (PI)													.256 (.032)	.000	.194	.318
Self-Efficacy (SE)													.634 (.032)	.000	.573	.696
Constant	4.048 (.062)	.000	3.927	4.169	5.305 (.059)	.000	5.189	5.421	5.165 (.054)	.000	5.059	5.271	-1.375 (.189)	.000	-1.747	-1.004
Conditional Indirect Effect via PMN																
DS[1] (0)													-.297 (.046)		-.390	-.210
DS (1)													-.140 (.036)		-.215	-.072
Conditional Indirect Effect via PI																
DS (0)													-.246 (.044)		-.335	-.165
DS (1)													.015 (.020)		-.024	.055
Conditional Indirect Effect via SE																
DS (0)													-.320 (.067)		-.453	-.192
DS (1)													.145 (.042)		.063	.228
R^2	.195				.160				.201				.423			
df	3.000				3.000				3.000				4.000			
F (*p*)	158.059	.000			124.629	.000			163.933	.000			359.293	.000		

[1]0= Inactive donors, 1=active donors
[2] “It is likely that I will donate blood in the future.”

Table S16. Detailed results of the moderated mediation analysis on donation intentions in the *long term* regarding pre-pandemic and pandemic (*t=1*).

	Personal Moral Norms				Perceived Impact				Self-Efficacy				Donation Intention[2]			
	b (se)	*p*	2.5%	97.5%	b (se)	*p*	2.5%	97.5%	b (se)	*p*	2.5%	97.5%	b (se)	*p*	2.5%	97.5%
Pandemic Effect	-.509 (.052)	.000	-.612	-.407	-.484 (.049)	.000	-.581	-.387	-.262 (.045)	.000	-.349	-.174	-.011 (.050)	.821	-.109	.087
Donor Status (DS)	1.029 (.088)	.000	-.857	1.201	.694 (.083)	.000	.531	.857	1.046 (.075)	.000	.899	1.193				
Pandemic Effect × DS	.236 (.084)	.005	.071	.401	.470 (.080)	.000	.313	.626	.310 (.072)	.000	.168	.451				
Personal Moral Norms (PMN)													.437 (.030)	.000	.378	.496
Perceived Impact (PI)													.141 (.033)	.000	.076	.205
Self-Efficacy (SE)													.502 (.034)	.000	.435	.568
Constant	4.048 (.062)	.000	3.927	4.169	5.305 (.059)	.000	5.190	5.420	5.165 (.053)	.000	5.061	5.269	-1.775 (.201)	.000	-2.168	-1.381
Conditional Indirect Effect via PMN																
DS[1] (0)													-.223 (.032)		-.288	-.164
DS (1)													-.119 (.030)		-.180	-.063
Conditional Indirect Effect via PI																
DS (0)													-.068 (.018)		-.105	-.035
DS (1)													.002 (.007)		-.016	.011
Conditional Indirect Effect via SE																
DS (0)													-.131 (.029)		-.191	-.075
DS (1)													.024 (.020)		-.016	.065
R^2	.184				.147				.191				.367			
df	3.000				3.000				3.000				4.000			
F (*p*)	135.730	.000			104.014	.000			141.809	.000			261.491	.000		

[1]0= Inactive donors, 1=active donors
[2] "I intend to donate blood on the next possible date."

Table S17. Detailed results of the moderated mediation analysis on donation intentions in the *short term* regarding pre-pandemic and pandemic (*t=2*).

	Personal Moral Norms				Perceived Impact				Self-Efficacy				Donation Intention[2]			
	b (se)	*p*	2.5%	97.5%	b (se)	*p*	2.5%	97.5%	b (se)	*p*	2.5%	97.5%	b (se)	*p*	2.5%	97.5%
Pandemic Effect	-.509 (.052)	.000	-.612	-.407	-.484 (.049)	.000	-.581	-.387	-.262 (.045)	.000	-.349	-.174	-.126 (.052)	.015	-.227	.025
Donor Status (DS)	1.029 (.088)	.000	-.857	1.201	.694 (.083)	.000	.531	.857	1.046 (.075)	.000	.899	1.193				
Pandemic Effect × DS	.236 (.084)	.005	.071	.401	.470 (.080)	.000	.313	.626	.310 (.072)	.000	.168	.451				
Personal Moral Norms (PMN)													.394 (.031)	.000	.333	.456
Perceived Impact (PI)													.191 (.034)	.000	.124	.257
Self-efficacy (SE)													.576 (.035)	.000	.507	.644
Constant	4.048 (.062)	.000	3.927	4.169	5.305 (.059)	.000	5.190	5.420	5.165 (.053)	.000	5.061	5.269	-1.766 (.207)	.000	-2.173	-1.360
Conditional Indirect Effect via PMN																
DS[1] (0)													-.201 (.030)		-.264	-.146
DS (1)													-.108 (.027)		-.163	-.056
Conditional Indirect Effect via PI																
DS (0)													-.092 (.020)		-.135	-.056
DS (1)													-.003 (.009)		-.021	.014
Conditional Indirect Effect via SE																
DS (0)													-.151 (.033)		-.218	-.087
DS (1)													.028 (.023)		-.018	.073
R^2	.184				.147				.191				.389			
df	3.000				3.000				3.000				4.000			
F (*p*)	135.730	.000			104.014	.000			141.809	.000			287.815	.000		

[1]0= Inactive donors, 1=active donors
[2] "I intend to donate blood over the next six months."

Table S18. Detailed results of the moderated mediation analysis on donation intentions in the *medium term* regarding pre-pandemic and pandemic (*t=2*).

	Personal Moral Norms				Perceived Impact				Self-Efficacy				Donation Intention[2]			
	b (se)	*p*	2.5%	97.5%	b (se)	*p*	2.5%	97.5%	b (se)	*p*	2.5%	97.5%	b (se)	*p*	2.5%	97.5%
Pandemic Effect	-.509 (.052)	.000	-.612	-.407	-.484 (.049)	.000	-.581	-.387	-.262 (.045)	.000	-.349	-.174	-.245 (.051)	.000	-.345	-.146
Donor Status (DS)	1.029 (.088)	.000	-.857	1.201	.694 (.083)	.000	.531	.857	1.046 (.075)	.000	.899	1.193				
Pandemic Effect × DS	.236 (.084)	.005	.071	.401	.470 (.080)	.000	.313	.626	.310 (.072)	.000	.168	.451				
Personal Moral Norms (PMN)													.273 (.031)	.000	.213	.333
Perceived Impact (PI)													.237 (.033)	.000	.171	.302
Self-Efficacy (SE)													.639 (.034)	.000	.571	.706
Constant	4.048 (.062)	.000	3.927	4.169	5.305 (.059)	.000	5.190	5.420	5.165 (.053)	.000	5.061	5.269	-1.197 (.204)	.000	-1.597	-.798
Conditional Indirect Effect via PMN																
DS[1] (0)													-.139 (.025)		-.193	-.094
DS (1)													-.075 (.020)		-.116	-.037
Conditional Indirect Effect via PI																
DS (0)													-.115 (.023)		-.163	-.073
DS (1)													-.003 (.011)		-.026	.018
Conditional Indirect Effect via SE																
DS (0)													-.167 (.037)		-.241	-.096
DS (1)													.031 (.026)		-.020	.081
R^2	.184				.147				.191				.401			
df	3.000				3.000				3.000				4.000			
F (*p*)	135.730	.000			104.014	.000			141.809	.000			301.666	.000		

[1]0= Inactive donors, 1=active donors
[2] "It is likely that I will donate blood in the future."

Table S19. Detailed results of the moderated mediation analysis on donation intentions in the *long term* regarding pre-pandemic and pandemic (*t=2*).

	Personal Moral Norms				Perceived Impact				Self-Efficacy				Donation Intention[2]			
	b (se)	*p*	2.5%	97.5%	b (se)	*p*	2.5%	97.5%	b (se)	*p*	2.5%	97.5%	b (se)	*p*	2.5%	97.5%
Pandemic Effect	-.380 (.037)	.000	-.452	-.309	-.340 (.034)	.000	-.407	-.272	-.184 (.031)	.000	-.246	-.123	.018 (.035)	.616	-.051	.087
Donor Status (DS)	1.029 (.088)	.000	.856	1.202	.694 (.083)	.000	.532	.857	1.046 (.075)	.000	.898	1.193				
Pandemic Effect × DS	.171 (.060)	.005	.053	.289	.335 (.057)	.000	.224	.446	.234 (.051)	.000	.133	.335				
Personal Moral Norms (PMN)													.427 (.031)	.000	.367	.487
Perceived Impact (PI)													.137 (.034)	.000	.071	.203
Self-Efficacy (SE)													.528 (.034)	.000	.460	.595
Constant	4.048 (.062)	.000	3.926	4.170	5.305 (.059)	.000	5.190	5.420	5.165 (.053)	.000	5.061	5.270	-1.856 (.203)	.000	-2.255	-1.458
Conditional Indirect Effect via PMN																
DS[1] (0)													-.162 (.022)		-.207	-.121
DS (1)													-.089 (.022)		-.133	-.045
Conditional Indirect Effect via PI																
DS (0)													-.047 (.013)		-.073	-.024
DS (1)													-.001 (.005)		-.010	.008
Conditional Indirect Effect via SE																
DS (0)													-.097 (.022)		-.142	-.055
DS (1)													.026 (.016)		-.005	.056
R^2	.191				.148				.191				.373			
df	3.000				3.000				3.000				4.000			
F (*p*)	136.475	.000			100.222	.000			136.313	.000			257.676	.000		

[1]0= Inactive donors, 1=active donors
[2] "I intend to donate blood on the next possible date."

Table S20. Detailed results of the moderated mediation analysis on donation intentions in the *short term* regarding pre-pandemic and pandemic (*t=3)*.

	Personal Moral Norms				Perceived Impact				Self-Efficacy				Donation Intention[2]			
	b (se)	*p*	2.5%	97.5%	b (se)	*p*	2.5%	97.5%	b (se)	*p*	2.5%	97.5%	b (se)	*p*	2.5%	97.5%
Pandemic Effect	-.380 (.037)	.000	-.452	-.309	-.340 (.034)	.000	-.407	-.272	-.184 (.031)	.000	-.246	-.123	-.112 (.036)	.002	-.183	-.041
Donor Status (DS)	1.029 (.088)	.000	.856	1.202	.694 (.083)	.000	.532	.857	1.046 (.075)	.000	.898	1.193				
Pandemic Effect × DS	.171 (.060)	.005	.053	.289	.335 (.057)	.000	.224	.446	.234 (.051)	.000	.133	.335				
Personal Moral Norms (PMN)													.376 (.031)	.000	.315	.438
Perceived Impact (PI)													.174 (.035)	.000	.106	.242
Self-Efficacy (SE)													.603 (.035)	.000	.534	.672
Constant	4.048 (.062)	.000	3.926	4.170	5.305 (.059)	.000	5.190	5.420	5.165 (.053)	.000	5.061	5.270	-1.743 (.209)	.000	-2.152	-1.333
Conditional Indirect Effect via PMN																
DS[1] (0)													-.143 (.021)		-.186	-.105
DS (1)													-.079 (.020)		-.119	-.040
Conditional Indirect Effect via PI																
DS (0)													-.059 (.014)		-.088	-.035
DS (1)													-.001 (.006)		-.013	.011
Conditional Indirect Effect via SE																
DS (0)													-.111 (.025)		-.162	-.063
DS (1)													.030 (.018)		-.006	.064
R^2	.191				.148				.191				.397			
df	3.000				3.000				3.000				4.000			
F (*p*)	136.475	.000			100.222	.000			136.313	.000			284.953	.000		

[1]0= Inactive donors, 1=active donors
[2] "I intend to donate blood over the next six months."

Table S21. Detailed results of the moderated mediation analysis on donation intentions in the *medium term* regarding pre-pandemic and pandemic (*t=3*).

	Personal Moral Norms				Perceived Impact				Self-Efficacy				Donation Intention[2]			
	b (se)	*p*	2.5%	97.5%	b (se)	*p*	2.5%	97.5%	b (se)	*p*	2.5%	97.5%	b (se)	*p*	2.5%	97.5%
Pandemic Effect	-.380 (.037)	.000	-.452	-.309	-.340 (.034)	.000	-.407	-.272	-.184 (.031)	.000	-.246	-.123	-.217 (.036)	.000	-.287	-.147
Donor Status (DS)	1.029 (.088)	.000	.856	1.202	.694 (.083)	.000	.532	.857	1.046 (.075)	.000	.898	1.193				
Pandemic Effect × DS	.171 (.060)	.005	.053	.289	.335 (.057)	.000	.224	.446	.234 (.051)	.000	.133	.335				
Personal Moral Norms (PMN)													.276 (.031)	.000	.216	.337
Perceived Impact (PI)													.218 (.034)	.000	.152	.285
Self-Efficacy (SE)													.656 (.035)	.000	.588	.724
Constant	4.048 (.062)	.000	3.926	4.170	5.305 (.059)	.000	5.190	5.420	5.165 (.053)	.000	5.061	5.270	-1.207 (.305)	.000	-1.609	-.805
Conditional Indirect Effect via PMN																
DS[1] (0)													-.105 (.018)		-.142	-.073
DS (1)													-.058 (.015)		-.089	-.029
Conditional Indirect Effect via PI																
DS (0)													-.074 (.016)		-.108	-.046
DS (1)													-.001 (.007)		-.016	.013
Conditional Indirect Effect via SE																
DS (0)													-.121 (.027)		-.176	-.069
DS (1)													.033 (.019)		-.006	.069
R^2	.191				.148				.191				.418			
df	3.000				3.000				3.000				4.000			
F (*p*)	136.475	.000			100.222	.000			136.313	.000			310.788	.000		

[1]0= Inactive donors, 1=active donors
[2] "It is likely that I will donate blood in the future."

Table S22. Detailed results of the moderated mediation analysis on donation intentions in the *long term* regarding pre-pandemic and pandemic (*t*=3).

	Personal Moral Norms				Perceived Impact				Self-Efficacy				Donation Intention[2]			
	b (se)	*p*	2.5%	97.5%	b (se)	*p*	2.5%	97.5%	b (se)	*p*	2.5%	97.5%	b (se)	*p*	2.5%	97.5%
Pandemic Effect	-.283 (.028)	.000	-.337	-.229	-.273 (.026)	.000	-.324	-.223	-.171 (.024)	.000	-.217	-.124	-.036 (.027)	.181	-.089	.017
Donor Status (DS)	1.029 (.088)	.000	.857	1.201	.694 (.083)	.000	.533	.856	1.046 (.075)	.000	.898	1.193				
Pandemic Effect × DS	.168 (.047)	.000	.076	.259	.295 (.044)	.000	.208	.381	.189 (.040)	.000	.111	.268				
Personal Moral Norms (PMN)													.429 (.031)	.000	.369	.489
Perceived Impact (PI)													.160 (.033)	.000	.095	.225
Self-Efficacy (SE)													.491 (.034)	.000	.425	.558
Constant	4.048 (.062)	.000	3.926	4.170	5.305 (.058)	.000	5.191	5.420	5.165 (.053)	.000	5.061	5.269	-1.787 (.206)	.000	-2.191	-1.383
Conditional Indirect Effect via PMN																
DS[1] (0)													-.122 (.017)		-.155	-.090
DS (1)													-.050 (.017)		-.084	-.016
Conditional Indirect Effect via PI																
DS (0)													-.044 (.010)		-.064	-.025
DS (1)													.003 (.004)		-.005	.012
Conditional Indirect Effect via SE																
DS (0)													-.084 (.016)		-.115	-.053
DS (1)													.009 (.010)		-.012	.029
R^2	.194				.161				.202				.379			
df	3.000				3.000				3.000				4.000			
F (*p*)	136.217	.000			109.265	.000			143.987	.000			259.154	.000		

[1]0= Inactive donors, 1=active donors
[2] “I intend to donate blood on the next possible date.”

Table S23. Detailed results of the moderated mediation analysis on donation intentions in the *short term* regarding pre-pandemic and pandemic (*t=4*).

	Personal Moral Norms				Perceived Impact				Self-Efficacy				Donation Intention[2]			
	b (se)	*p*	2.5%	97.5%	b (se)	*p*	2.5%	97.5%	b (se)	*p*	2.5%	97.5%	b (se)	*p*	2.5%	97.5%
Pandemic Effect	-.283 (.028)	.000	-.337	-.229	-.273 (.026)	.000	-.324	-.223	-.171 (.024)	.000	-.217	-.124	-.107 (.028)	.000	-.161	-.053
Donor Status (DS)	1.029 (.088)	.000	.857	1.201	.694 (.083)	.000	.533	.856	1.046 (.075)	.000	.898	1.193				
Pandemic Effect × DS	.168 (.047)	.000	.076	.259	.295 (.044)	.000	.208	.381	.189 (.040)	.000	.111	.268				
Personal Moral Norms (PMN)													.383 (.032)	.000	.322	.445
Perceived Impact (PI)													.210 (.034)	.000	.143	.276
Self-Efficacy (SE)													.567 (.035)	.000	.498	.635
Constant	4.048 (.062)	.000	3.926	4.170	5.305 (.058)	.000	5.191	5.420	5.165 (.053)	.000	5.061	5.269	-1.769 (.211)	.000	-2.183	-1.356
Conditional Indirect Effect via PMN																
DS[1] (0)													-.109 (.016)		-.140	-.080
DS (1)													-.044 (.016)		-.075	-.014
Conditional Indirect Effect via PI																
DS (0)													-.057 (.011)		-.081	-.037
DS (1)													.005 (.006)		-.007	.016
Conditional Indirect Effect via SE																
DS (0)													-.097 (.018)		-.132	-.062
DS (1)													.010 (.012)		-.014	.033
R^2	.194				.161				.202				.409			
df	3.000				3.000				3.000				4.000			
F (*p*)	136.217	.000			109.265	.000			143.987	.000			293.992	.000		

[1]0= Inactive donors, 1=active donors
[2] “I intend to donate blood over the next six months.”

Table S24. Detailed results of the moderated mediation analysis on donation intentions in the *medium term* regarding pre-pandemic and pandemic (*t=4*).

	Personal Moral Norms				Perceived Impact				Self-Efficacy				Donation Intention[2]			
	b (se)	*p*	2.5%	97.5%	b (se)	*p*	2.5%	97.5%	b (se)	*p*	2.5%	97.5%	b (se)	*p*	2.5%	97.5%
Pandemic Effect	-.283 (.028)	.000	-.337	-.229	-.273 (.026)	.000	-.324	-.223	-.171 (.024)	.000	-.217	-.124	-.172 (.027)	.000	-.225	-.119
Donor Status (DS)	1.029 (.088)	.000	.857	1.201	.694 (.083)	.000	.533	.856	1.046 (.075)	.000	.898	1.193				
Pandemic Effect × DS	.168 (.047)	.000	.076	.259	.295 (.044)	.000	.208	.381	.189 (.040)	.000	.111	.268				
Personal Moral Norms (PMN)													.279 (.031)	.000	.219	.340
Perceived Impact (PI)													.268 (.034)	.000	.202	.334
Self-Efficacy (SE)													.625 (.034)	.000	.558	.692
Constant	4.048 (.062)	.000	3.926	4.170	5.305 (.058)	.000	5.191	5.420	5.165 (.053)	.000	5.061	5.269	-1.325 (.208)	.000	-1.732	-.917
Conditional Indirect Effect via PMN																
DS[1] (0)													-.079 (.014)		-.107	-.055
DS (1)													-.032 (.011)		-.055	-.010
Conditional Indirect Effect via PI																
DS (0)													-.073 (.013)		-.100	-.049
DS (1)													.006 (.007)		-.009	.020
Conditional Indirect Effect via SE																
DS (0)													-.107 (.020)		-.146	-.068
DS (1)													.011 (.013)		-.015	.037
R^2	.194				.161				.202				.431			
df	3.000				3.000				3.000				4.000			
F (*p*)	136.217	.000			109.265	.000			143.987	.000			322.176	.000		

[1]0= Inactive donors, 1=active donors
[2] "It is likely that I will donate blood in the future."

Table S25. Detailed results of the moderated mediation analysis on donation intentions in the *long term* regarding pre-pandemic and pandemic (*t=4*).

	Personal Moral Norms				Perceived Impact				Self-Efficacy				Donation Intention[2]			
	b (se)	*p*	2.5%	97.5%	b (se)	*p*	2.5%	97.5%	b (se)	*p*	2.5%	97.5%	b (se)	*p*	2.5%	97.5%
Pandemic Effect	-.200 (.023)	.000	-.245	-.155	-.191 (.022)	.000	-.233	-.149	-.108 (.019)	.000	-.146	-.070	-.012 (.022)	.602	-.055	.032
Donor Status (DS)	1.029 (.088)	.000	.857	1.201	.694 (.083)	.000	.532	.857	1.046 (.074)	.000	.900	1.192				
Pandemic Effect × DS	.099 (.039)	.011	.023	.175	.197 (.037)	.000	.126	.269	.123 (.033)	.000	.059	.188				
Personal Moral Norms (PMN)													.453 (.031)	.000	.391	.514
Perceived Impact (PI)													.140 (.034)	.000	.073	.207
Self-Efficacy (SE)													.511 (.035)	.000	.440	.579
Constant	4.048 (.062)	.000	3.926	4.170	5.305 (.059)	.000	5.190	5.420	5.165 (.053)	.000	5.062	5.268	-1.885 (.211)	.000	-2.298	-1.472
Conditional Indirect Effect via PMN																
DS[1] (0)													-.090 (.014)		-.120	-.065
DS (1)													-.046 (.015)		-.076	-.016
Conditional Indirect Effect via PI																
DS (0)													-.027 (.007)		-.042	-.014
DS (1)													.001 (.003)		-.006	.008
Conditional Indirect Effect via SE																
DS (0)													-.055 (.013)		-.081	-.030
DS (1)													.008 (.009)		-.011	.026
R^2	.172				.135				.186				.374			
df	3.000				3.000				3.000				4.000			
F (*p*)	115.173	.000			86.799	.000			126.291	.000			248.515	.000		

[1]0= Inactive donors, 1=active donors
[2] "I intend to donate blood on the next possible date."

Table S26. Detailed results of the moderated mediation analysis on donation intentions in the *short term* regarding pre-pandemic and pandemic (*t=5*).

	Personal Moral Norms				Perceived Impact				Self-Efficacy				Donation Intention[2]			
	b (se)	*p*	2.5%	97.5%	b (se)	*p*	2.5%	97.5%	b (se)	*p*	2.5%	97.5%	b (se)	*p*	2.5%	97.5%
Pandemic Effect	-.200 (.023)	.000	-.245	-.155	-.191 (.022)	.000	-.233	-.149	-.108 (.019)	.000	-.146	-.070	-.083 (.023)	.000	-.128	-.039
Donor Status (DS)	1.029 (.088)	.000	.857	1.201	.694 (.083)	.000	.532	.857	1.046 (.074)	.000	.900	1.192				
Pandemic Effect × DS	.099 (.039)	.011	.023	.175	.197 (.037)	.000	.126	.269	.123 (.033)	.000	.059	.188				
Personal Moral Norms (PMN)													.396 (.032)	.000	.334	.459
Perceived Impact (PI)													.186 (.035)	.000	.117	.254
Self-Efficacy (SE)													.594 (.036)	.000	.523	.665
Constant	4.048 (.062)	.000	3.926	4.170	5.305 (.059)	.000	5.190	5.420	5.165 (.053)	.000	5.062	5.268	-1.851 (.215)	.000	-2.273	-1.430
Conditional Indirect Effect via PMN																
DS[1] (0)													-.079 (.013)		-.106	-.056
DS (1)													-.040 (.013)		-.067	-.014
Conditional Indirect Effect via PI																
DS (0)													-.036 (.008)		-.053	-.021
DS (1)													.001 (.005)		-.008	.010
Conditional Indirect Effect via SE																
DS (0)													-.064 (.015)		-.094	-.035
DS (1)													.009 (.011)		-.013	.030
R^2	.172				.135				.186				.404			
df	3.000				3.000				3.000				4.000			
F (*p*)	115.173	.000			86.799	.000			126.291	.000			281.441	.000		

[1]0= Inactive donors, 1=active donors
[2] “I intend to donate blood over the next six months.”

Table S27. Detailed results of the moderated mediation analysis on donation intentions in the *medium term* regarding pre-pandemic and pandemic (*t=5*).

	Personal Moral Norms				Perceived Impact				Self-Efficacy				Donation Intention[2]			
	b (se)	*p*	2.5%	97.5%	b (se)	*p*	2.5%	97.5%	b (se)	*p*	2.5%	97.5%	b (se)	*p*	2.5%	97.5%
Pandemic Effect	-.200 (.023)	.000	-.245	-.155	-.191 (.022)	.000	-.233	-.149	-.108 (.019)	.000	-.146	-.070	-.147 (.022)	.000	-.191	-.103
Donor Status (DS)	1.029 (.088)	.000	.857	1.201	.694 (.083)	.000	.532	.857	1.046 (.074)	.000	.900	1.192				
Pandemic Effect × DS	.099 (.039)	.011	.023	.175	.197 (.037)	.000	.126	.269	.123 (.033)	.000	.059	.188				
Personal Moral Norms (PMN)													.280 (.032)	.000	.218	.341
Perceived Impact (PI)													.236 (.035)	.000	.168	.304
Self-Efficacy (SE)													.636 (.036)	.000	.566	.706
Constant	4.048 (.062)	.000	3.926	4.170	5.305 (.059)	.000	5.190	5.420	5.165 (.053)	.000	5.062	5.268	-1.208 (.212)	.000	-1.625	-.792
Conditional Indirect Effect via PMN																
DS[1] (0)													-.056 (.010)		-.078	-.037
DS (1)													-.028 (.010)		-.048	-.010
Conditional Indirect Effect via PI																
DS (0)													-.045 (.010)		-.065	-.028
DS (1)													.002 (.006)		-.010	.012
Conditional Indirect Effect via SE																
DS (0)													-.069 (.016)		-.101	-.038
DS (1)													.010 (.012)		-.014	.032
R^2	.172				.135				.186				.412			
df	3.000				3.000				3.000				4.000			
F (*p*)	115.173	.000			86.799	.000			126.291	.000			290.954	.000		

[1]0= Inactive donors, 1=active donors

[2] "It is likely that I will donate blood in the future."

Table S28. Detailed results of the moderated mediation analysis on donation intentions in the *long term* regarding pre-pandemic and pandemic (*t=5)*.

	Personal Moral Norms				Perceived Impact				Self-Efficacy				Donation Intention[2]			
	b (se)	*p*	2.5%	97.5%	b (se)	*p*	2.5%	97.5%	b (se)	*p*	2.5%	97.5%	b (se)	*p*	2.5%	97.5%
Pandemic Effect	-.231 (.020)	.000	-.270	-.192	-.162 (.019)	.000	-.199	-.124	-.119 (.017)	.000	-.152	-.085	.001 (.020)	.965	-.038	.040
Donor Status (DS)	1.029 (.087)	.000	.859	1.199	.694 (.082)	.000	.534	.855	1.046 (.074)	.000	.901	1.191				
Pandemic Effect × DS	.166 (.034)	.000	.100	.233	.167 (.032)	.000	.103	.230	.098 (.029)	.000	.041	.155				
Personal Moral Norms (PMN)													.478 (.033)	.000	.414	.542
Perceived Impact (PI)													.097 (.036)	.007	.027	.167
Self-Efficacy (SE)													.500 (.036)	.000	.429	.572
Constant	4.048 (.061)	.000	3.928	4.168	5.305 (.058)	.000	5.192	5.419	5.165 (.052)	.000	5.063	5.268	-1.708 (.215)	.000	-2.131	-1.286
Conditional Indirect Effect via PMN																
DS[1] (0)													-.110 (.014)		-.138	-.084
DS (1)													-.031 (.031)		-.060	-.003
Conditional Indirect Effect via PI																
DS (0)													-.016 (.006)		-.028	-.005
DS (1)													.001 (.002)		-.004	.005
Conditional Indirect Effect via SE																
DS (0)													-.059 (.012)		-.083	-.037
DS (1)													-.010 (.009)		-.029	.007
R^2	.214				.131				.194				.375			
df	3.000				3.000				3.000				4.000			
F (*p*)	145.703	.000			80.316	.000			128.623	.000			239.855	.000		

[1]0= Inactive donors, 1=active donors
[2] "I intend to donate blood on the next possible date."

Table S29. Detailed results of the moderated mediation analysis on donation intentions in the *short term* regarding pre-pandemic and pandemic (*t*=6*)*.

	Personal Moral Norms				Perceived Impact				Self-Efficacy				Donation Intention[2]			
	b (se)	*p*	2.5%	97.5%	b (se)	*p*	2.5%	97.5%	b (se)	*p*	2.5%	97.5%	b (se)	*p*	2.5%	97.5%
Pandemic Effect	-.231 (.020)	.000	-.270	-.192	-.162 (.019)	.000	-.199	-.124	-.119 (.017)	.000	-.152	-.085	-.072 (.021)	.001	-.112	-.031
Donor Status (DS)	1.029 (.087)	.000	.859	1.199	.694 (.082)	.000	.534	.855	1.046 (.074)	.000	.901	1.191				
Pandemic Effect × DS	.166 (.034)	.000	.100	.233	.167 (.032)	.000	.103	.230	.098 (.029)	.000	.041	.155				
Personal Moral Norms (PMN)													.416 (.034)	.000	.350	.483
Perceived Impact (PI)													.130 (.037)	.000	.058	.202
Self-Efficacy (SE)													.586 (.038)	.000	.512	.660
Constant	4.048 (.061)	.000	3.928	4.168	5.305 (.058)	.000	5.192	5.419	5.165 (.052)	.000	5.063	5.268	-1.581 (.222)	.000	-2.017	-1.146
Conditional Indirect Effect via PMN																
DS[1] (0)													-.096 (.013)		-.123	-.072
DS (1)													-.027 (.013)		-.052	-.003
Conditional Indirect Effect via PI																
DS (0)													-.021 (.006)		-.035	-.009
DS (1)													.001 (.003)		-.005	.006
Conditional Indirect Effect via SE																
DS (0)													-.069 (.014)		-.096	-.044
DS (1)													-.012 (.011)		-.034	.008
R^2	.214				.131				.194				.395			
df	3.000				3.000				3.000				4.000			
F (*p*)	145.703	.000			80.316	.000			128.623	.000			261.306	.000		

[1]0= Inactive donors, 1=active donors
[2] "I intend to donate blood over the next six months."

Table S30. Detailed results of the moderated mediation analysis on donation intentions in the *medium term* regarding pre-pandemic and pandemic (*t=6)*.

	Personal Moral Norms				Perceived Impact				Self-Efficacy				Donation Intention[2]			
	b (se)	*p*	2.5%	97.5%	b (se)	*p*	2.5%	97.5%	b (se)	*p*	2.5%	97.5%	b (se)	*p*	2.5%	97.5%
Pandemic Effect	-.231 (.020)	.000	-.270	-.192	-.162 (.019)	.000	-.199	-.124	-.119 (.017)	.000	-.152	-.085	-.114 (.020)	.000	-.154	-.074
Donor Status (DS)	1.029 (.087)	.000	.859	1.199	.694 (.082)	.000	.534	.855	1.046 (.074)	.000	.901	1.191				
Pandemic Effect × DS	.166 (.034)	.000	.100	.233	.167 (.032)	.000	.103	.230	.098 (.029)	.000	.041	.155				
Personal Moral Norms (PMN)													.293 (.033)	.000	.229	.358
Perceived Impact (PI)													.195 (.036)	.000	.125	.266
Self-Efficacy (SE)													.645 (.037)	.000	.573	.717
Constant	4.048 (.061)	.000	3.928	4.168	5.305 (.058)	.000	5.192	5.419	5.165 (.052)	.000	5.063	5.268	-1.093 (.217)	.000	-1.519	-.666
Conditional Indirect Effect via PMN																
DS[1] (0)													-.068 (.012)		-.091	-.046
DS (1)													-.019 (.009)		-.037	-.002
Conditional Indirect Effect via PI																
DS (0)													-.032 (.008)		-.048	-.018
DS (1)													.001 (.004)		-.007	.009
Conditional Indirect Effect via SE																
DS (0)													-.076 (.015)		-.106	-.048
DS (1)													-.014 (.012)		-.037	.008
R^2	.214				.131				.194				.411			
df	3.000				3.000				3.000				4.000			
F (*p*)	145.703	.000			80.316	.000			128.623	.000			278.911	.000		

[1]0= Inactive donors, 1=active donors
[2] "It is likely that I will donate blood in the future."

Table S31. Detailed results of the moderated mediation analysis on donation intentions in the *long term* regarding pre-pandemic and pandemic (*t=6*).

	Active Donors<br>Model 1<br>Short-term donation intentions			Inactive Donors<br>Model 2<br>Short-term donation intentions		
Predictors	β	CI	*p*	β	CI	*p*
**Self-efficacy**	**.21**	**.09-.34**	**.001**	**.08**	**.04-.13**	**<.001**
**Personal moral norms**	**.14**	**.03-.25**	**.015**	**.08**	**.03-.13**	**.002**
**Perceived impact**	**.16**	**.03-.28**	**.012**	-.02	-.05-.02	.366
Concern	-.02	-.11-.07	.663	.02	-.02-.07	.340
Expected return to everyday life	-.08	-.18-.02	.100	-.02	-.07-.03	.510
**Informed**	**.17**	**.10-.25**	**<.001**	.01	-.02-.05	.410
Trust in COVID-19 measures	.07	-.09-.22	.416	.05	-.01-.11	.110
SARS-CoV-2 infection[a]	-.44	-1.61-0.72	.454	.80	-.37-1.96	.179
**Blood donation frequency**	**.49**	**.07-.90**	**.021**			
Time period fixed effects	Yes			Yes		
No. of observations	888			1863		
No. of observations used	654			1412		
R^2	.118			.023		

Table S32. Panel regression analysis for *active* and *inactive donors* relying on the unbalanced panel.
First-difference estimator. Significant results are marked in bold. [a] (1: yes / 0: no).
